# New chlorophylls designed by theoretical spectroscopy and machine learning

**DOI:** 10.1007/s11120-025-01183-0

**Published:** 2025-12-11

**Authors:** Fabian Weber, Simon Petry, Dennis J. Nürnberg, Jan P. Götze

**Affiliations:** 1 Freie Universität Berlin, Fachbereich Biologie Chemie Pharmazie, Physikalische und Theoretische Chemie, Arnimallee 22, 14195 Berlin, Germany; 2 Freie Universität Berlin, Fachbereich Physik, Experimentelle Biophysik, Arnimallee 14, 14195 Berlin, Germany; 3 Freie Universität Berlin, Dahlem Centre of Plant Sciences, Albrecht-Thaer-Weg 6, 14195 Berlin, Germany

**Keywords:** Chlorophyll, Machine learning, Density functional theory, UV/vis spectroscopy

## Abstract

The main pigment for oxygenic photosynthesis, chlorophyll (Chl) *a*, is structurally related to several other Chl variants, naturally occurring mostly with mono-oxidized substitutions. These include Chl *b*, Chl *d* and Chl *f* and divinyl chlorophylls (DVChls) *a* and *b*. In this contribution, we computationally explore an expanded set of over 250,000 Chl variants, looking for potentially interesting targets for synthetic biology. We focus on optical properties, employing a machine learning (ML) approach and subsequently verifying the corresponding predictions using time-dependent density functional theory (TD-DFT) and multireference DFT (DFT/MRCI). We find that (i) Chl *f* is the best monosubstituted red-shifted Chl, as no other Chl in our set exceeds Chl *f* in terms of both red shift and absorption intensity, (ii) Chl *b* is not the best Chl to harvest photons from the green region of the optical spectrum, as several other Chls with the same or better green absorbance were identified (most notably DVChl *b*) and (iii) the T_1_ energy of Chls can be slightly adapted. The latter would enable experiments to determine whether it is beneficial having the T_1_ transition energy located between the two lowest O_2_ singlet state transitions, as it is found for Chl *a*. This might be a prerequisite for stable, efficient oxygen generation. Our ML approach thus provides a comprehensive overview on an extensive subset of potential Chl modifications that could be used for tuning oxygenic photosynthesis, if suitable synthesis pathways can be found.

**Supplementary information:**

The online version contains supplementary material available at 10.1007/s11120-025-01183-0.

## Introduction

**Fig. 1:**
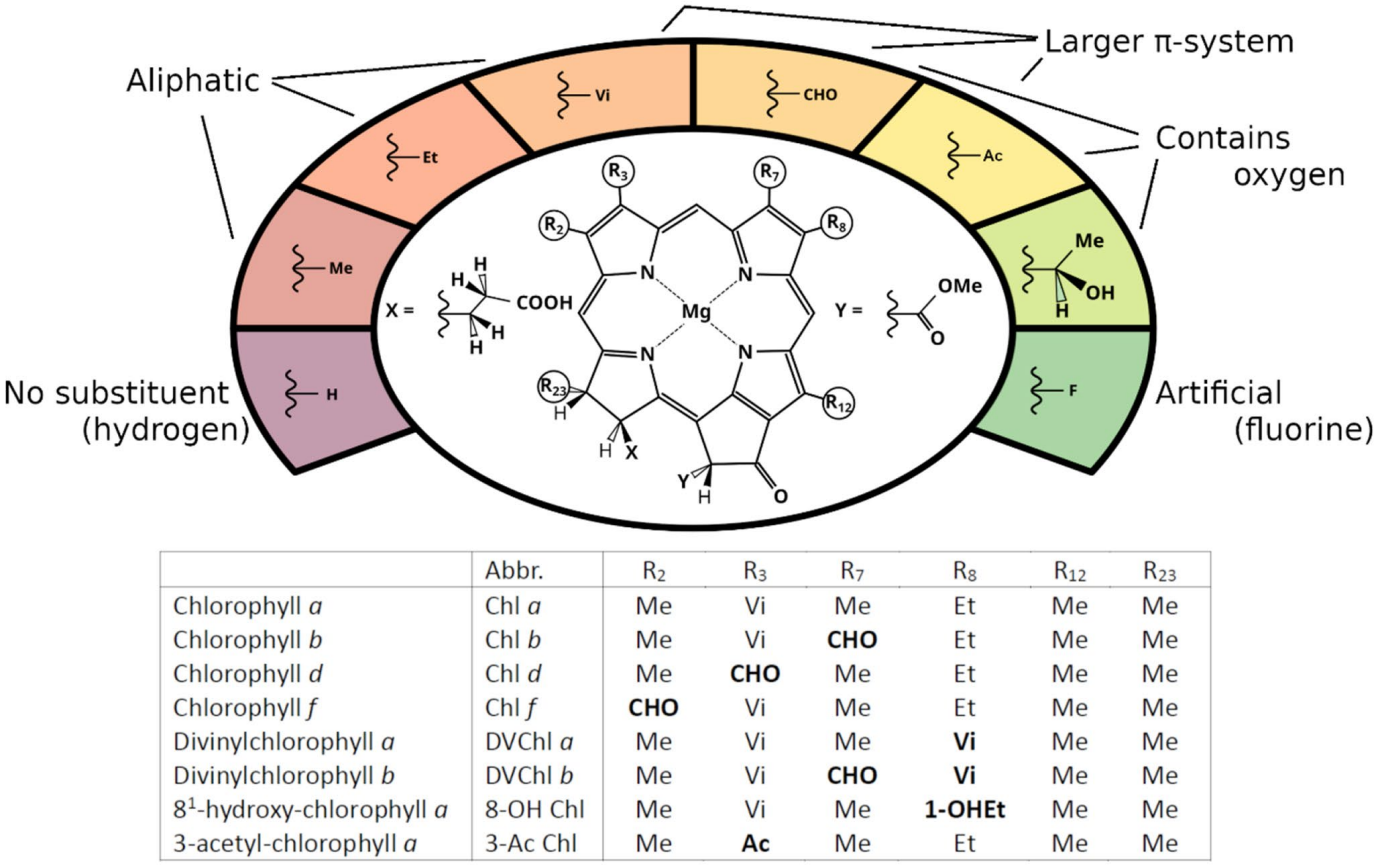
Chemical core structure for the chl variant set (ChlVS), chosen test substitution sites (R_n_, with n being the connected carbon atom number) and the tested substituents: (i) no substituent (H), (ii) aliphatic (Me(thyl), Et(hyl), Vi(nyl)), (iii) mono-oxidized (CHO (i.e., formyl), Ac(etyl), 1-OHEt (i.e., 1-hydroxyethyl)) and (iv) an artificial substituent (fluorine, F). Hypothetically, 8^6 = 262,144 chl variants would be possible. Chl models were built as shown, with residue X replacing the phytyl chain. The bottom list indicates the members of our ChlVS that are naturally occurring (or, for 3-Ac Chl, experimentally characterized), with the differences to Chl *a* highlighted in bold

Chlorophyll (Chl) *a* is the main pigment of oxygenic photosynthesis, due to its comparatively strong redox potential when forming a charge transfer (CT) state in a Chl *a* dimer (Ishikita et al. 2005), with recent findings constantly addressing the corresponding donor/acceptor roles (Sirohiwal and Pantazis 2023; Müller et al. 2010). In most photosynthetic systems, Chl *a* also forms the basis for excitation energy transfer (EET), which fuels the CT formation process, and further is strongly absorbing throughout the ultraviolet/visible (UV/vis) range of solar irradiation. Combined with a relatively long-lived excited state (Niedzwiedzki and Blankenship 2010), Chl *a* provides all features that allowed for the evolution of the photosynthetic apparatus (Lokstein et al. 2021). Recently, however, the naturally occurring Chl pigments found in photosynthetic complexes were considered to be less than optimal for agricultural applications regarding photosynthetic efficiency (Elias et al. 2024, 2021; Wang et al. 2025; Chen and Blankenship 2011; Ort et al. 2015). Several variants of Chl *a* exist, which are derived from various steps of the Chl *a* synthesis pathway (Myśliwa-Kurdziel et al. 2019; Chen 2019; Rüdiger 2002; Grimm et al., 2006; Bryant et al. 2020). Aside from the large family of bacteriochlorophylls, for which the evolutionary background still remains unclear (Larkum 2006; Nishihara et al. 2024), several major Chl derivates are known and well-characterized: Chl *b* (Tanaka et al. 1998), the family of Chl *c* pigments (Büchel 2020), Chl *d* and Chl *f* (Chen 2019); as well as the divinyl-(DV)Chls *a* and *b* (Steglich et al. 2003). Aside from Chl *d*, all of these Chl variants show a slightly larger π-system than Chl *a*. Chls *b*, *d* and *f* feature a single additional oxygen atom in comparison to Chl *a*. An overview of the Chl variants relevant for this study is provided in Fig. 1, bottom list. Despite the often slightly larger π-conjugated systems, the resulting UV/vis spectra are not simply shifted to lower energies: Some state transition energies are higher (blue shift), while some are lower (red shift). This is due to the type and position of the chemical modifications, specific for each Chl variant, and is possibly best illustrated by comparing the drastically different spectra of Chl *b* and *f* (see Fig. 3 for a visual comparison).

Several studies have aimed to elucidate the corresponding structure-spectrum relationship based on the natural Chl varieties (Graczyk et al. 2014; Götze et al. 2022). It was found that the orbitals of the involved Chl excitations are very close to the porphyrin picture by Gouterman (1961), with Chl *a* actually adhering less to this idealized scheme than the more intensely modified variants (e.g., the Chl *c*-type family). Following this four-orbital model (forming the excited states via the two highest occupied and lowest unoccupied orbitals), the expectation for Chls is to present two sets of optical excitations, namely the lower energy, “red” Q band and a higher energy, “blue” B band (or “Soret” band). Of the two, the B band should have a higher total absorption intensity. The studies further showed that the employed methods suffice for the intended calculations, which leads to our choice of quantum chemical methodology as outlined later in this article.

Although chemically possible, the full set of Chl *a* substitutions is not realized in nature. Chl variants are likely only kept when beneficial for the organism, and due to the toxicity of free Chls (Damaraju et al. 2011), their benefit is likely restricted to the context of photosynthesis. Because Chl *a* is synthesized from natural amino acids (Bryant et al. 2020), their original constitution poses a limiting factor to the (bio-)chemical set that is currently observed. Post-synthesis modifications are also often limited to local mono-oxidation, as indicated by Chl *b*, *d*, *f* or 8-OH Chl (Fig. 1).

Recent developments in artificial enzyme evolution and synthesis via organic chemistry, however, open up the possibility of expanding the Chl set beyond what is naturally available (Bunzel et al. 2021; Hanreich et al. 2023; Liu et al. 2018). Here, we explore the spectroscopic properties of currently not realized Chl variants. Due to the large size of the possible Chl variant set (ChlVS, see Fig. 1, top, for our chosen subset of sites and substituents), we employ machine learning (ML) to perform a virtual screening and characterization for Chls that are viable candidates in the context of a synthetic biology approach. Their potential usefulness will be evaluated below on the basis of the predicted optical spectra, as well as the structural similarity to existing Chl variants, to possibly simplify their (bio-)chemical realization.

From the plethora of potentially interesting Chl modifications, we chose to perform the following spectroscopic changes: (i) A red shift of the energetically lower Chl states (Q band), (ii) a red shift of the energetically higher Chl states (B band) or (iii) a change in the lowest Chl triplet state energy while maintaining the corresponding singlet excitation. Before discussing the results and properties of the predicted ChlVS, we will shortly outline the computational approach, with focus on ML used for facilitating the virtual screening.

## Methods

### Molecular structures

To reduce computational effort, the ChlVS did not include the phytyl chain, which was truncated as shown in Fig. 1. The ChlVS core structure (Fig. 1, top and section S1 in the SI) encompasses several of the naturally occurring Chls (Fig. 1, bottom). We thus have seven experimentally characterized variants to test our predictions. The chosen substitution sites of the ChlVS result from the strategic decision to allow for most of the naturally occurring substitutions (at R_2_, R_3_, R_7_ and R_8_), as well as two additional sites located at the structural opposite ends (at R_12_ and R_23_). The substituent set encompasses all residues found in the natural variants (methyl, ethyl, vinyl, formyl and 1-hydroxyethyl in the biologically found stereochemistry), the possibility for their absence (hydrogen), an only artificially realized (Smith and Calvin 1966), but logical extension of the natural set (acetyl) and an unusual member (fluorine). Fluorine is especially interesting due to recent developments in corresponding organic synthesis strategies (Tironi et al. 2020), which would allow for modification of Chls into new variants that are currently unavailable in nature. C-F bonds are controversial due to their high stability, and we thus wanted to test if F could potentially be beneficial or detrimental to the desired Chl properties.

### ML model

The generation of the ChlVS database and training for all of the nine ML models (one for each property: five state energies, resulting in the four presented excitation energies as their differences, and four oscillator strengths) used in this study was performed within the ArchOnML program package (Weber 2025a, b). The package manages and organizes quantum chemistry (QC) calculations, with the aim to train Kernel Ridge Regression (KRR) models, that predict the properties of interest from computationally inexpensive (semi-empirical quantum chemistry, SQC) descriptors. KRR models present an adequate choice for the screening of finite chemical sets (such as a ChlVS), since a single model can be trained on most standard devices within minutes of computation time. The trade-off is that there exists practically no transferability to other core/substituent combinations. While training a multi-purpose deep neural network may allow for such transferability, it is not required for the problem at hand.

The overall procedure of a screening study comprises three main steps, namely, training data generation, model training and predictions. A pictorial representation can be found in the SI, section S6, Figure S1. The first step can be further broken down in the following calculations: (a) Generation of initial guess structures, (b) structural SQC optimization (“pre-optimization”, using the PM6 method) (Stewart 2007), with the optimized structure serving as reference single point for the descriptors (c) density functional theory (DFT) structure optimization (using the CAM-B3LYP method with a 6-31 G* basis) (Yanai et al. 2004; Hariharan and Pople 1973), and (d) time-dependent (TD-)DFT (Casida et al. 1998; Jamorski et al. 1996; Casida and Salahub 2000) to obtain excited state properties (same method and basis as for (c)). The last step (d) is not a required part of the general ArchOnML procedure, but specific for the present article. Further, only the training data will be subject to (a) to (d) for obtaining the ML model, while the rest of the ChlVS will only be treated with steps (a) and (b).

The label values of the first triplet excitation energy ($\Delta {E_{0 \to T1}}$) were obtained by replacing (d) with a ground state calculation (multiplicity = 3) and taking the energy difference to the singlet ground state energies. All (S)QC calculations during the above procedure were performed with the Gaussian16 program package (Frisch et al. 2016). Here, several different SQC methods are available; PM6 was selected, since previous work using this method showed success (Weber and Mori 2022). Future studies focusing on the ML method may benchmark the different performances with respect to the selected SQC method. Details on the initial guess structure generator of ArchOnML can be found in the respective documentation (Weber 2025b) and in section S12 of the SI. We chose to omit implicit solvents for the present study, to increase computational efficiency. Since the aim is to provide an initial assessment of the ChlVS, we only present implicit solvent calculations only for a few select candidates: The SI (section S8) contains the spectra of the highest-interest Chl variants found in this study in implicit acetone. Our results further show (Fig. 3) that the gas phase predictions align well with the relative energies found in solvation: For the Chls that are experimentally characterized, this underlines the validity of gas phase calculations in terms of relative energy order.

To then train the KRR model, the Chl training data was split into randomized training and testing sets with a 4:1 ratio. Because of wavefunction convergence issues, mostly for highly unrealistic structures/substitution patterns leading to steric clashes/overcrowded regions (determined by visual inspection), the original training set size of 8,000 molecules was slightly reduced (9 in step (b), 74 in step (c), and 16 in step (d)) before splitting the training and testing sets. For the triplets specifically, the convergence in step (d) was significantly worse than for TD-DFT, resulting in 2,051 molecules dropping out of the set prior to splitting. This is likely because the singlet ground state structure was unsuitable to converge a triplet wavefunction. Therefore, 4,755 (1,194) molecules remained in its training (testing) sets for the triplet energy model, while all singlet state properties contained 6,320 (1,581) molecules. Since KRR models contain hyperparameters that require optimization outside of the actual training approach, a grid-based, stratified five-fold cross-validation scheme was applied to identify the best, mean hyperparameters (SI, section S2). Ultimately, one individual model was trained in this manner for each target property (SI, section S3) – where stratification of the training data was performed with respect to the respective target property of choice. Further details are provided in the corresponding software documentation (Weber 2025b), and via input examples found in the SI, section S10.

After finishing all calculations for the training set, the label values were extracted from the outputs of the QC calculations. Here, the excitation energies $\Delta E$ and oscillator strengths $f$ of the singlet-singlet transitions required an additional distinction criterion: The Q_y_ and Q_x_ states (the lowest excited singlet states S_1_ and S_2_, respectively, usually with transitions below 2.5 eV/500 nm) could simply be identified as the energetically ordered first and second TD-DFT-computed singlet states. In contrast, extracting the properties of B band states (with transitions higher than 2.5 eV/500 nm) turned out to be more difficult, because several states were usually found in close proximity in this higher energy region. Hence, the energetically ordered indices of the QC outputs do not necessarily point to states of the same excitation character when comparing different Chls. To avoid training on insufficiently characterized states, a distinction scheme was applied that extracts the two brightest (highest $f$) excited states from that energetic region and then groups them based on their energetic ordering. This extraction scheme was motivated by the assumption that the characteristic π-π* transitions of the B_x_ and B_y_ states (typically found among S_3_, S_4_ and S_5_) would stand out in terms of $f$. A more detailed explanation of how the extraction criterion was implemented is given in the SI, section S7. We would also like to point out that we first aimed to characterize the states by means of their transition dipole moment orientations; however, such a scheme was found to be similarly affected by mixing of energetically close states. It was ultimately discarded in favor of the approach based only on $f$.

To finalize the training, ArchOnML was used to generate all descriptors and train the KRR models for each of the studied properties. The exact settings used for grid-based hyperparameter optimizations can be found in the SI, section S2. After confirming that the properties can indeed be trained to a satisfactory degree (see section 3.1), the structures of the remaining ChlVS were generated with ArchOnML and subjected to the same SQC optimization as the training set. For the prediction set, no further DFT optimization is needed, since the desirable quantities will be predicted on the basis of descriptors in SQC quality. This forms the reason for the expected speed-up (see sec. 3.1) compared to conventionally calculating everything in DFT quality. Out of the 254,144 molecules to predict, 3,284 were unable to converge their SQC optimization calculation. These unsuccessful candidates were not considered further and discarded from the ChlVS.

### Calculations for selected ChlVS candidates

To further substantiate the predictions generated by the ML model, we performed TD-DFT calculations on the newly proposed Chl variants found in this study, using the same methods as listed under the section “ML model” (CAM-B3LYP/6-31 G* both for structural optimizations and standalone TD-DFT) (Yanai et al. 2004; Hariharan and Pople 1973). We further performed DFT-based multireference configuration interaction (DFT/MRCI (Kleinschmidt et al. 2009; Lyskov et al. 2016)) calculations utilizing the SVP basis set (Schäfer et al. 1992), to provide comparisons for the TD-DFT values. Triplet states were obtained by optimizing the molecular structures with a spin multiplicity of three. These calculations were all performed using the ORCA QC software package (Neese 2022), also some cases employing a continuum solvent model (CPCM) as indicated in the results section (Cossi et al. 2003).

## Results and discussion

### Training performance of the ML model

Table 1 summarizes the performance of each of the ML models in terms of the mean absolute error (MAE), coefficient of determination (r^2^) and double standard deviation of prediction errors $2{\sigma _{err}}$, when predicting the properties of a test set excluded from training. The latter quantity gives an impression of the largest expected error, assuming a corresponding normal distribution.

**Table 1:** Model performance on predicting absolute chl variant energies $\Delta {E_{0 \to X}}$ and oscillator strengths $f$, presenting the mean absolute errors (MAEs), coefficient of determination (r^2^) and the (doubled) standard deviation of normal distribution 2${\sigma _{err}}$

Target state	$\Delta {E_{0 \to X}}$			$f$		
	MAE/eV	r^2^	$2{\sigma _{err}}$/eV	MAE	r^2^	$2{\sigma _{err}}$
Q_y_	0.012	0.947	0.025	0.009	0.938	0.020
Q_x_	0.016	0.944	0.031	0.006	0.939	0.009
B_x_	0.020	0.938	0.038	0.062	0.864	0.114
B_y_	0.025	0.912	0.061	0.084	0.372	0.144
T_1_	0.020	0.919	0.044	n/a	n/a	n/a

Regarding the excitation energies, the $\Delta {E_{0 \to X}}$ values of all considered states can be predicted by the model within at least a $2{\sigma _{err}}$ of roughly 0.06 eV. Since we were only interested in relative changes between Chl variants, and because of the expected absolute, intrinsic (TD)-DFT error of 0.1–0.3 eV (Laurent and Jacquemin 2013), we considered the models to be sufficiently accurate. Further, all coefficients of determination are at least above 0.91, attesting to a high confidence in the predicted values. Note that the performances of the Q_y_ and Q_x_ states are, however, slightly better than for the two B-band states, which is likely caused by the intermixing of close-lying states in the B band and the resulting variance in the corresponding state characters, as discussed above.

The problem with state impurities is further attested by the ML performance when predicting oscillator strengths $f$. The KRR model directly relies on wavefunction information of the SQC calculation (Weber 2025a, b; Weber and Mori 2022). From Table 1 it can be seen that the Q band states’ oscillator strengths are indeed reproduced in the ML testing with satisfactory confidence (r^2^ values above 0.93). However, $f\left( {{B_x}} \right)$ can be reproduced with only an r^2^ of 0.86, and the corresponding value for $f\left( {{B_y}} \right)$ is very low (0.37), making predictions regarding B_y_ unreliable. Since $f$ is directly dependent on the transition character (i.e. transition overlap integrals), our chosen extraction scheme can successfully identify B_x_, but not B_y_. The reason for this is likely that we typically identified a singular, exceptionally bright state in the B band region (i.e., B_x_). This state is then accompanied by several less intense ones – out of which the brightest one was arbitrarily assigned to B_y_. Table 1 thus shows that consistently selecting the state with the highest $f$ value results in a relatively consistent B_x_ state assignment. However, it also shows that this does not apply for the second-highest state, making a more elaborate character analysis necessary when including B_y_. Since it is also unclear if all Chls actually exhibit two or more bright B band states (Götze et al. 2022; Sirohiwal et al. 2020), we therefore decided to omit B_y_ from any further detailed analysis and continued using the state selection criteria described above – keeping a skeptic stance towards the relatively low quality of $f\left( {{B_y}} \right)$ predictions.

**Fig. 2:**
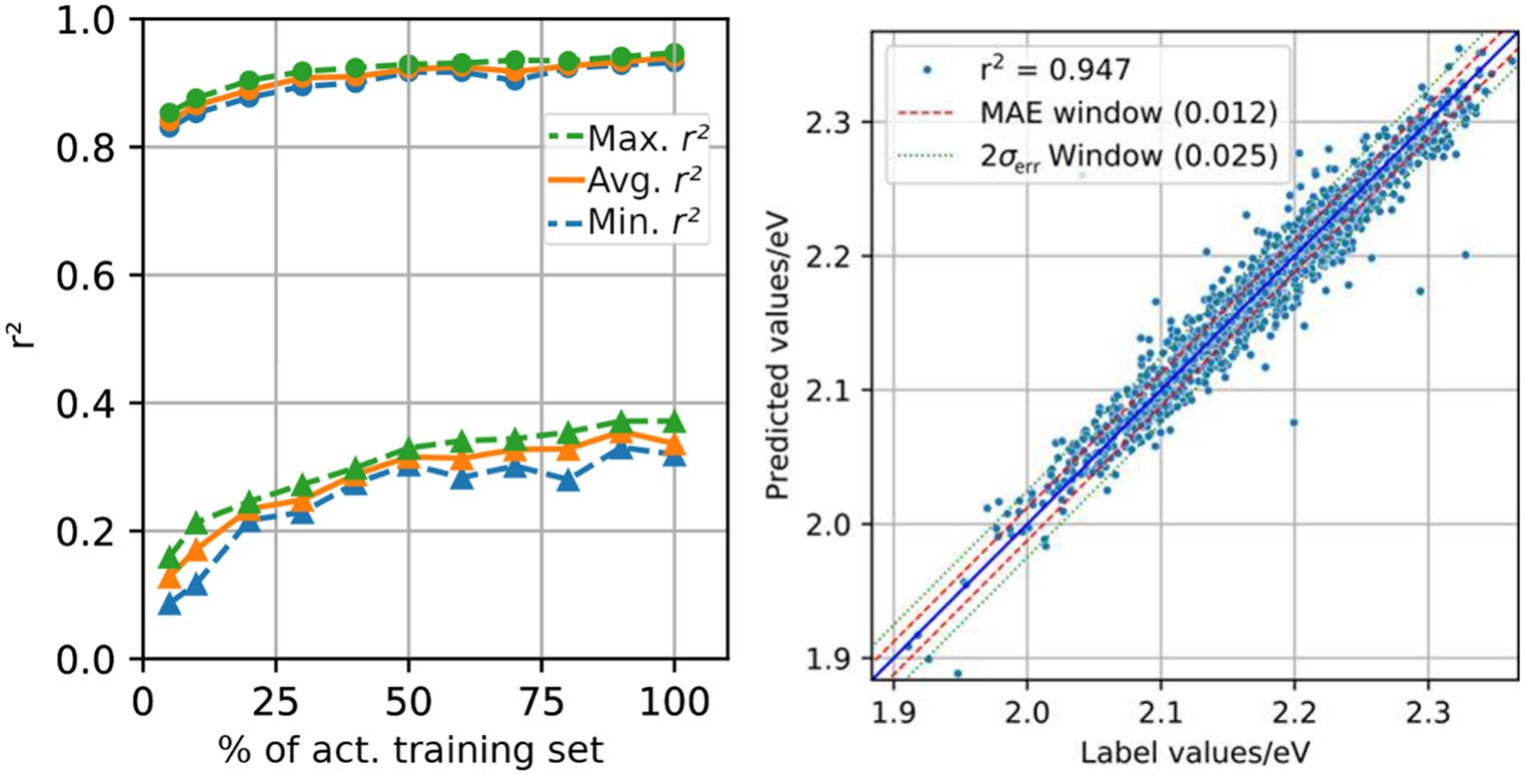
Selected ML model quality criteria. Left: best (●) and worst (▲) training scenarios encountered in this study; circles: training for $\Delta {E_{0 \to S1}}$; triangles: $f\left( {{B_y}} \right)$. Detailed curves can be found in the SI, section S3. The dashed curves represent the highest and lowest encountered performances at each individual percentage of training set throughout all considered individual curves. Right: correlation diagram of the values predicted by the $\Delta {E_{0 \to S1}}$ model against the actual label values for the testing data, that was not part of the training procedure. Related data can be found in the SI, sections S4 and S5

Finally, to check the robustness of the models, Fig. 2, left, shows mean learning curves for the properties $\Delta {E_{0 \to S1}}$ and $f\left( {{B_y}} \right)$, which represent the best and worst performing models obtained in this study, respectively. Here, the average learning curve refers to the mean performance of five models that have been trained on differently selected sets of training and testing molecules each time (see SI, section 3, for individual curves).

As can be seen for the $\Delta {E_{0 \to S1}}$ models (Fig. 2, left), the coefficient of determination does not improve beyond 30% of the available training data. The spread between the best and worst model within the models tested for $\Delta {E_{0 \to S1}}$, however, does further decrease until 90% – indicating that the model still became more robust with more training data. Despite the small divergence between lowest and highest performance at 100% of active training data (80% of the full training set, as noted above), the final model for predictions was constructed from the full training data set in all cases. A correlation diagram of the predicted and actual test label values of this final model can be seen in Fig. 2, right.

For the $f\left( {{B_y}} \right)$ model (Fig. 2, left), the r^2^ value will likely improve further if more training data were added, since there is still a pronounced positive slope when approaching the 100% mark. Note that this is likely a result of the state impurity as well: If the model is presented with molecules that display a total of N states of different character, the training would likely require close to N times the amount of training data to achieve a comparable performance to a pure state model. However, due to the mixing of state character at different geometries/substitution patterns, it might very well be that there is an accuracy ceiling to the combined descriptors in the KRR model, with r^2^ possibly never exceeding a (unknown) limit. For $f\left( {{B_y}} \right)$, the individual learning curves also display a slight overfitting behavior (see SI, section S3), since the curves start to diverge at around 50%. Since other oscillator strength models are not showing overfitting issues (see SI, section S3), we assume that this is also an effect of the impure state characters, not affecting the other predicted properties.

**Fig. 3:**
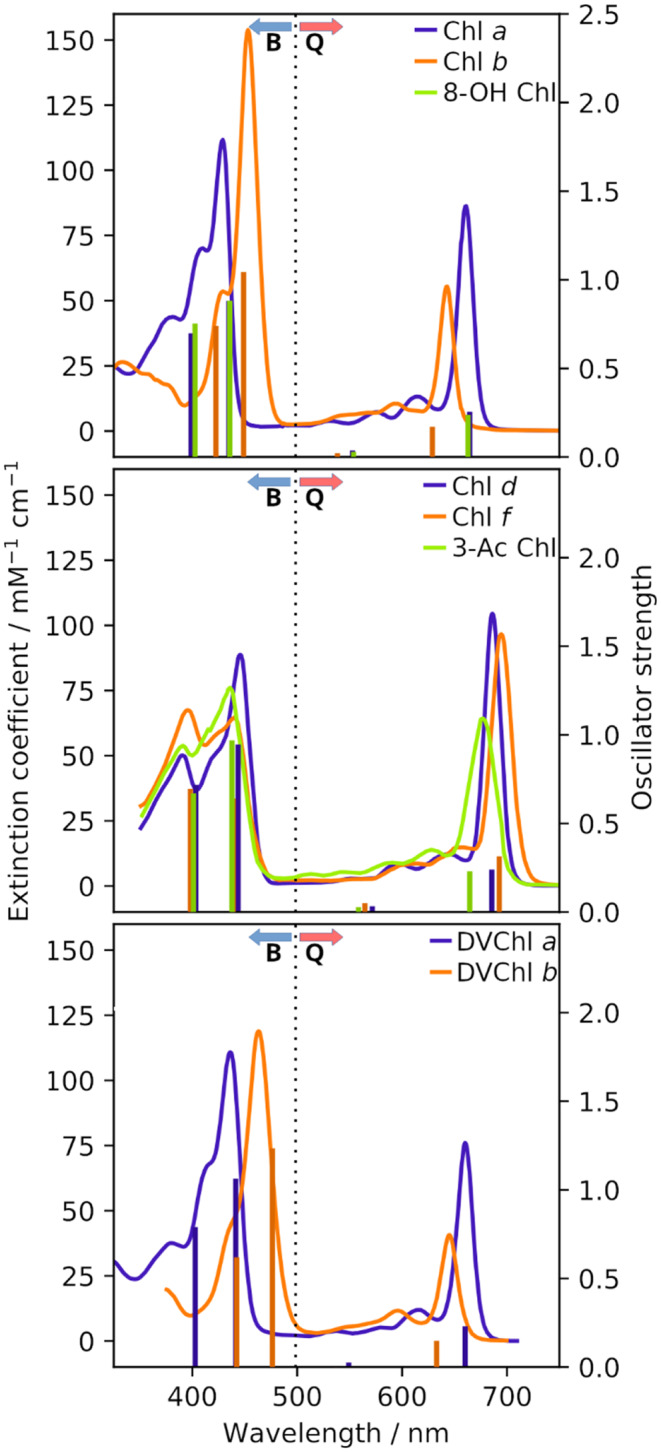
Experimental optical spectra (in acetone, lines), as well as the four ML predicted vertical excitation energies and oscillator strengths (bars) of various Chls. See section S11, Table S5 in the SI for detailed values. Predicted Q (B) band energies were shifted by −0.278 (−0.615) eV to obtain the displayed graphs. Experimental data from the given sources (Steglich et al. 2003; Smith and Calvin 1966; Strain et al. 1963; Taniguchi and Lindsey 2018; Goedheer 1966; Li et al. 2012; Shedbalkar and Rebeiz 1992). Q and B band regions indicated by dotted line/arrows. The experimental Chl *a* and 8-OH Chl (not shown) spectra are virtually identical

Our ChlVS also contains the experimentally well-characterized Chl variants depicted in Fig. 1. The corresponding comparison to our predictions can be found in Fig. 3. Q and B band predictions had to be shifted with strongly different values to roughly match the experimental maxima: Averaging over the different pigments yields “experiment minus prediction” energy shifts of −0.278 eV (Q band) and −0.615 eV (B band). Regardless of the absolute error, the observed order of predicted Q and B band energies corresponds well to the experimental order (e.g., Q band energies, Chl *b* > Chl *a* > Chl *d* > Chl *f*). We do observe, however, that some of our predicted DVChl B band energies are too low in energy, after shifting, while the Q_y_ Chl *b*, DVChl *b* and 3-Ac Chl excitation energies are slightly too high. We have investigated this issue and found that the corresponding residues are likely not well represented: They are prone to become co-planar with the Chl chlorin ring structure after geometry optimization. Crystal structures of Chls (e.g., when bound in proteins, but without any direct interaction to the vinyl residue) show that this is not realistic (Su et al. 2017). Instead, vinyl groups are twisted with respect to the chlorin ring, likely due to steric hindrance of nearby methyl or ethyl groups. While this does not have a substantial effect on the common Chls (or we correct for this implicitly via the shifting), the DVChls may become outliers. As such, we refrain in some cases from discussing vinyl and acetyl in-depth for Q band energy changes, since the corresponding Q_y_ energy differences between the Chls were found to be much less than those for the B band (and thus might be significantly affected by the planarity of our optimized geometries).

Finally, comparing the average conventional calculation CPU time (SQC pre-optimization, DFT-optimization, TD-DFT excited state calculation) and their full prediction time (SQC pre-optimization, SQC single-point, prediction time) of the training set yields an average speed-up factor of 490 (data not shown). In the following, we exploit this massively increased computational efficiency to predict potentially improved Chl variants.

### Chls with likely better performance in the roles of Chls d and f

Natural Chl variants absorb in different spectral regions, allowing photosynthetic organism to occupy various environmental niches (Sanfilippo et al. 2019). Some of these Chls, such as Chls *d* and *f,* allow growth above the previously assumed red-limit of photosynthesis at 700 nm (Elias et al. 2024). We therefore tested whether there are other Chls that perform this function in a better way, e.g., with higher absorbance or at even lower S_0_→Q_y_ energy, $\Delta {E_{0 - S1}}$. We did not restrict our $\Delta {E_{0 - S1}}$ values, although a low value may also result in redox potentials being too low for water splitting.

Of the full ChlVS, we identified 24,434/14,601 Chl variants that had a lower $\Delta {E_{0 - 1}}$ than Chl *d*/Chl *f*. This large difference is surprising given that our predicted Chl *d* and Chl *f* Q_y_ energies only differ by 0.02 eV, suggesting that we are reaching the upper limit of the ChlVS in terms of red-shifted Q_y_. Applying an oscillator strength criterion, i.e., taking only those entries that are at least as strongly absorbing as the predicted Chl *f* ($f$ = 0.31) and at equal or lower energies than Chl *f* (2.068 eV), resulted in 3,467 entries. Of those, only one is reachable from Chl *a* with a single modification: Chl *f*. This might imply that Chl *f* is the result of a biochemical optimization process, with Chl *d* possibly being an intermediate step or even evolutionary remnant, as it has a smaller red shift and less absorption intensity.

To get an even more red shifted Chl than Chl *f* is thus challenging. However, by introducing a second modification, we obtained nine molecules that could absorb at lower energies than Chl *f*. Within this group, four contain additional acetyl and vinyl residues, which we however omitted for the reasons outlined above. Eliminating those entries left us with five candidates, which all retain a formyl group at R_2_, as found in Chl *f*. Chl *f* thus forms a necessary basis but can be further red-shifted as shown in Table 2. The predicted red shifts are small (less than 10 nm compared to Chl *f*) and might exceed the accuracy limit of the TD-DFT calculations. However, being relative changes, we consider the shown trends and the relative order of changes to be trustworthy, even if the absolute values are likely not.

**Table 2:** Proposed single modifications to Chl *f* to obtain an improved red absorbing Chl Q band. Candidates with additional vinyl substitutions were excluded due to likely too low predicted $\Delta {E_{0 - S1}}$ values. Approximate nm shift relative to Chl *f* computed after correcting the computed energies by −0.278 eV for better overlap with experimental data. Corresponding TD-DFT and DFT/MRCI values can be found in the SI, sections S8 and S9

Chl *f* (R_2_: Formyl); add. modification	$\Delta \Delta {E_{0 - S1}}$/eV (to Chl *f*)	Approx. shift/nm (to Chl *f*)	$\Delta {f_{0 - S1}}$ (to Chl *f*)
R_3_: Formyl (“Chl *df*”)	−0.021	+8.2	+0.01
R_8_: Methyl	−0.005	+1.9	+0.01
R_12_: Ethyl	−0.004	+1.5	+0.02
R_12_: Formyl (“Chl *f+*”)	−0.023	+9.0	+0.05
R_23_: Hydrogen	−0.002	+0.8	+0.01

Two interesting candidates were identified as potential Chl *f* alternatives in the context of synthetic biology, designated here as “Chl *df*” and “Chl *f+*”. They both showed the largest red shift of the Q_y_ absorption by 8–9 nm in comparison to Chl *f*, while the other variants only showed a red shift of less than 2 nm (Table 2). Both variants introduce small red shifts to the Q_y_ absorption, while slightly increasing absorption, unless B band absorbance is also relevant for overall photon yield. In that case, Chl *df* and Chl *f+* are likely worse than their “parent” compounds (data not shown). Chl *df* features two structurally close oxygen atoms (at R_2_ and R_3_) in the Chl structure, and it could have also been the result of a rational design approach, as it simply combines the substitution patterns of features of Chls *d* and *f*. The two close oxygen atoms might, however, result in structural problems for synthesis and protein binding of the compound. Yet, given the possibility of dihedral rotation, the two formyl groups might not sterically hinder each other in the synthesized compound. It must be seen in practice if this poses an issue for synthesis of Chl *df* or its binding to proteins.

In contrast, Chl *f+* contains a change at the usually “inactive” R_12_ position, with a nearly identical result as found in Chl *df*. Structurally, both compounds feature changes at the same diametral chlorin axis, although at different ends (Chl *df*: R_3_, Chl *f+*: R_12_). This indicates that electron-withdrawing groups connected to the π-system at these locations result in the desired red shift with high absorption. Comparing Chl *df* to Chl *f+* would indicate that concentrating formyl groups at one end of the chlorin ring is (minutely) counterproductive for absorbance. This can be potentially explained by a slight intramolecular CT character of the state introduced by formyl groups concentrated at one side (R_2_ and R_3_), and CT states usually have lower absorbance.

### Chls with lower B band absorption energies

Aside from the Chls discussed in the previous section, which present a red shifted Q band, various natural Chls show a strong, red shifted B band. These Chls, such as Chl *b* and the Chl *c*-type family, also have a generally much stronger absorbance of their B bands, at the cost of the respective Q band absorption strength. We thus proposed in the past that the primary purpose of those compounds might rather be in the B band and shaping the interactions between the related states (Götze and Lokstein 2023). The generally more acknowledged explanation, however, is that a red shift of the B band would improve the overall absorption cross section (Kume et al. 2018).

Regardless of the actual biological role, a ChlVS for the red shifted B band can be built; analogous to the discussion of tuned Chls *d* and *f* presented above. Here, our reference compounds were Chl *a* and *b* (the strongly B band red shifted Chl *c*-type pigments are not available via our ChlVS core structure). We find that 139,324 tested cases, representing more than half of our total ChlVS, show a B band with lower energies than Chl *a*. Note the brightest B band state is usually the energetically lowest state of the B band (as a result of the ML training); this state was taken as the reference for this analysis. Our predicted Chl *b* bright B_x_ state is shifted by 0.089 eV to the respective state of Chl *a*, which is slightly less than expected from the corresponding maxima in experiment/acetone, for which 0.15 eV are found (. However, it is not as red-shifted as 77,005 other variants of the predicted ChlVS. As discussed earlier in this article, however, Chl *b* might simply be the (bio-)chemically accessible Chl for that role.

When requiring a B band absorption that is at least as high as Chl *b* ($f \ge$ 1.04), while absorbing at equal or lower energies ($\Delta {E_{0 - SX}} \le$ 3.378 eV, state X being state 3 in all relevant cases), 24,129 variants fulfill these criteria. However, there are only four variants which require a single change with respect to Chl *a*, including Chl *b* itself. The others are listed in Table 3, upper part, showing only minute improvements over Chl *b*. All of these monosubstituted Chl *a* variants, however, seem biologically accessible, with the exception of R_8_-acetyl. This would indicate that Chl *b* may not be as optimized as Chl *f*, or that it may also fulfill another role as noted in the beginning of this section (Götze and Lokstein 2023).

**Table 3:** Proposed single and double modifications to Chl *a* to obtain an improved red absorbing Chl B band compared to Chl *b*. Target state X is strongest absorbing B band state, found to be excited state 3 (in energetic order) for all relevant cases. Approximate nm shift compared to Chl *b* computed after correcting the computed energies by −0.615 eV for better overlap with experimental data. Corresponding TD-DFT and DFT/MRCI values can be found in the SI

Chl *a* modification	$\Delta \Delta {E_{0 - SX}}$/eV (to Chl *b*)	Approx. shift/nm (to Chl *b*)	$\Delta {f_{0 - SX}}$ (to Chl *b*)
R_7_: Vinyl	−0.008	+1.2	+0.02
R_8_: Formyl	−0.035	+5.4	+0.02
R_8_: Acetyl	−0.002	+0.3	+0.04
Chl *b* (R_7_: Formyl); add. modification	$\Delta \Delta {E_{0 - SX}}$/eV (to Chl *b*)	Approx. shift/nm (to Chl *b*)	$\Delta {f_{0 - SX}}$ (to Chl *b*)
R_2_: Hydrogen	−0.021	+3.2	+0.11
R_3_: Formyl (“Chl *bd*”)	−0.089	+14.1	+0.03
R_3_: Acetyl	−0.073	+11.5	+0.03
R_8_: Methyl	−0.002	+0.3	+0.00
R_8_: Vinyl (DVChl *b*)	−0.159	+25.8	+0.19
R_8_: Formyl (“Chl *bb*”)	−0.155	+25.1	+0.05
R_8_: Acetyl	−0.119	+19.1	+0.13
R_8_: ^1^EtOH	−0.087	+13.7	+0.06
R_12_: Ethyl	−0.004	+0.6	+0.01
R_23_: Ethyl	−0.000	+0.0	+0.04
R_23_: Vinyl	−0.024	+3.7	+0.08
R_23_: Formyl	−0.017	+2.6	+0.16
R_23_: Acetyl	−0.003	+0.5	+0.06

The space of related double modifications is much larger (47) than for the analogous Chl *f*/Q band case discussed above. Also, not all double modifications are based on the Chl *b* structure. Thus, a full discussion of all candidate structures would be excessive; we therefore only present those variants that seem to be chemically accessible as a single modification of Chl *b* (13 structures, Table 3, lower part).

The Chl *b* variants with red shifted B bands display a similar variety as found for the red shifted Chl *f*, with some of them being of particular interest. First and foremost, we correctly predict DVChl *b* to be a member of the set. However, the predicted DVChl *b* B band energies are found to be red shifted compared to the experiment (by about 0.07 eV, cf. Fig. 3). Furthermore, the impact on the B bands is smaller due to the overall larger shifts compared to the Q band, and we thus decided to keep the vinyl modifications (cf. the caveats outlined above). Out of the displayed Chl *b* modifications, DVChl *b* is found to have the strongest gain in absorbance and red shift. While the latter is likely overestimated by our model, it is however very interesting to see that virtually no other Chl *b* modification should exceed DVChl *b* in terms of absorbance in the blue/green spectral region. This would be corroborated by the observation that DVChl *b* is found in organisms with the corresponding light quality (Ito and Tanaka 2011; Barrera-Rojas et al. 2018). This pigment might thus be a viable candidate for replacement studies: If the role of Chl *b* is actually to “close the green gap” and nothing else, DVChl *b* should perform much better than Chl *b*. If not, as we proposed before (Götze and Lokstein 2023), replacing Chl *b* by DVChl *b* should be detrimental for the blue light resilience of the organism, when paired with normal Chl *a*. It should be noted that “closing the green gap” not only involves a red shifted B band, but also a blue shifted Q band. Experimentally, however, a blue shifted Q band usually absorbs far less than B. Q thus only plays a minor role for absorbance in the Chl green gap (in contrast to bacteriochlorophylls). The green gap can further be alleviated by Chl packing (Merzlyak et al. 2009), leading to the question why structurally modified Chls would be needed in the first place for this purpose.

Aside from DVChl *b*, several other candidates in Table 3 display a stronger B band red shift than found for Chl *b*, of which a few will be highlighted in the following. Position R_8_ can also be filled with a formyl group, which results in the compound we call “Chl *bb*”. This variant is predicted to have a B band red shift similar to that of DVChl *b*, yet with a slightly smaller gain in absorbance. However, it should be noted that as our model performs better for formyl groups, the B band red shift of Chl *bb* might actually be stronger than that of DVChl *b*. Alternatively, acetyl might be used at R_8_, but the shift is predicted to be smaller, but with a higher gain in absorbance compared to formyl. Analogously, we find that a formyl group at R_3_ provides a red shift in the B band, yet with a minute increase in absorbance. As this modification is naturally available (in Chl *d*), this compound might be biochemically more accessible, and we therefore term it “Chl *bd*”. If the introduction of DVChl *b in vivo* turns out to be difficult, Chls *bb* and *bd* might serve as viable alternatives that are chemically close to Chl *b*.

### Adapting Chl triplet energies

Chls are typically found assembled in tight clusters, which sometimes undergo intersystem crossing (ISC), especially under high light conditions. This can cause the formation of singlet oxygen, an undesired process that is highly dangerous to the organisms (Krieger-Liszkay 2004). The value of the Chl lowest triplet state energy might lead to degeneracy or close energies to the O_2_ T→S transition energies (about 0.98 and 1.64 eV), possibly enabling the process to run faster (cf. Fermi’s Golden Rule) (Kearns 1971). As such, it is interesting to locate Chl *a* in the ChlVS of possible Q band singlet/triplet pairs and see how it relates to other, potentially also accessible, Chl variants.

**Fig. 4:**
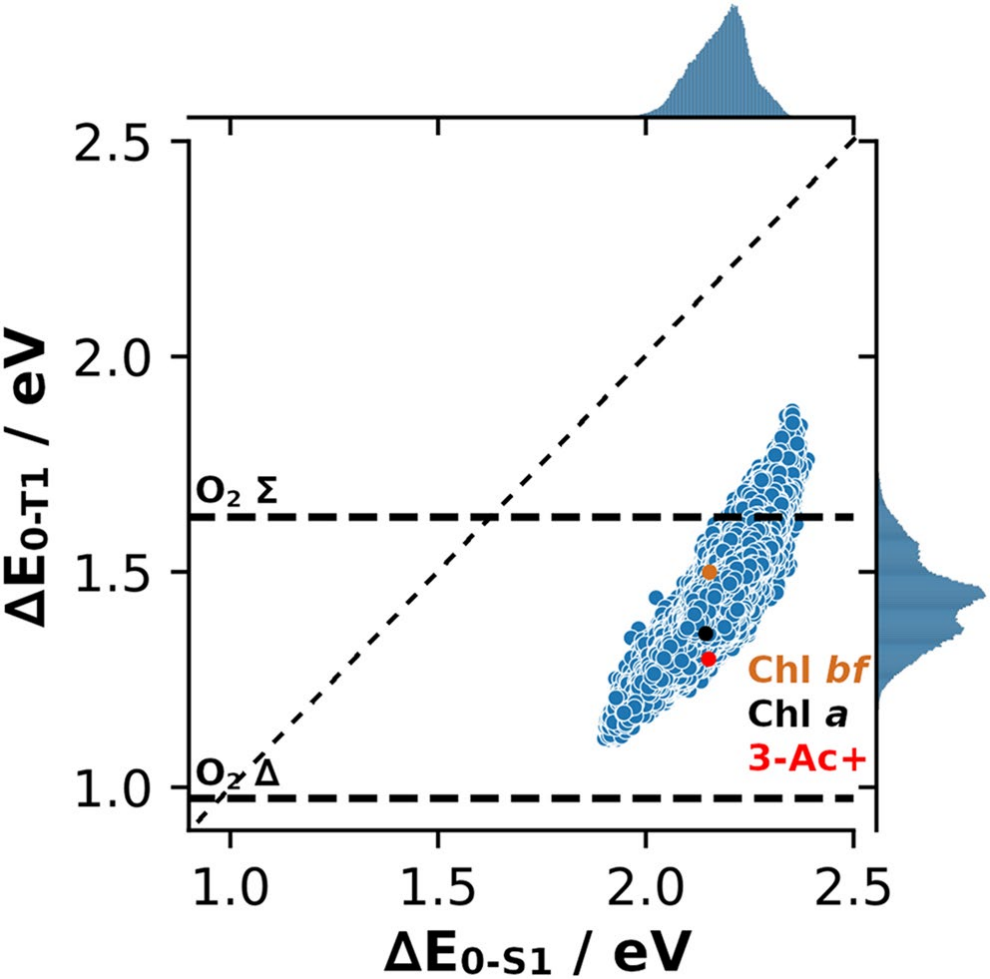
Overview on the full ChlVS, showing $\Delta {E_{0 - T1}}$ vs. $\Delta {E_{0 - S1}}$ for all computed cases. Singlet-to-triplet excitation energies of molecular oxygen also indicated as horizontal, dashed lines. Chl *a* highlighted, as well as two variants noted in the text

An overview of the Q_y_ singlet/triplet pairs can be found in Fig. 4. It indicates that there are no possible variants in our ChlVS that allow for inverted singlet/triplet energies (i.e., $\Delta {E_{0 - T1}} > \Delta {E_{0 - S1}}$ is never encountered). Since our ChlVS only introduces small changes to the system, and never affects the core chlorin ring structure, this result is not surprising. With higher energies, however, the ratio shifts slightly towards the diagonal, indicating that some higher energy variants (outside our ChlVS) might exhibit a state order inversion. Biologically though, these are likely not of interest, as the Chl *a* Q band energy is also tuned towards formation of the CT state required to perform the water splitting process in photosystem II (PSII). Shifting to much higher Q band energies might introduce unforeseeable issues with the PSII machinery, such as being able to reduce or oxidize other, “unintended” parts of the system.

On the other hand, the investigated singlet/triplet pairs show that, in relation to Chl *a*, there is potential to modify the triplet energies without much affecting $\Delta {E_{0 - S1}}$. The Chl *a* triplet energy (1.338 eV, after a correction shift of −0.019 eV) seems to be located almost perfectly in between the two O_2_ singlet excitations (energy gaps: −0.4 eV and 0.3 eV, respectively). We thus screened our set for Chls with nearly unchanged $\Delta {E_{0 - S1}}$ (less than 0.01 eV difference), while modifying Chl *a* as little as possible, aiming for a maximal change in $\Delta {E_{0 - T1}}$, either up or down in energies. In the set of Chls predicted to be within ±0.005 eV of $\Delta {E_{0 - S1}}\left( {Chla} \right)$, 21,232 ChlVS entries were identified. From those, we selected Chl variants that can be achieved with three modifications from Chl *a* and selected those that have the 10 highest/lowest $\Delta {E_{0 - T1}}$ (fewer modifications have a much lower T_1_ energy deviation from Chl *a*; data not shown). The results can be found in Table 4.

**Table 4:** Proposed triple modifications (and one double modification, italics) to Chl *a* to vary the triplet state excitation energy, while being conservative regarding $\Delta {E_{0 - S1}}$. Corresponding TD-DFT values can be found in the SI

Modifications for lower $\Delta {E_{0 - T1}}$	$\Delta \Delta {E_{0 - T1}}$/eV (to Chl *a*)	Modifications for higher $\Delta {E_{0 - T1}}$	$\Delta \Delta {E_{0 - T1}}$/eV (to Chl *a*)
R_2_: H; R_12_: formyl; R_23_: Et	−0.055	R_2_: Ac; R_3_: Me; R_8_: formyl	+0.119
R_2_: H; R_7_: F; R_12_: formyl	−0.064	R_2_: Ac; R_7_: 1-OHEt; R_8_: H	+0.110
R_2_: F; R_8_: Ac; R_12_: formyl	−0.061	R_2_: Ac; R_7_&R_8_: F	+0.137
R_3_&R_12_: formyl; R_8_: F	−0.073	R_2_: Ac; R_8_: formyl; R_23_: H	+0.126
R_3_: H; R_12_: formyl; R_23_: Vi	−0.057	R_2_&R_8_: Ac; R_23_: Et	+0.107
R_3_: Ac; R_7_: Et; R_8_: formyl	−0.075	R_2_&R_8_: Ac; R_23_: Vi	+0.118
R_3_: Ac; R_8_: 1-OHEt; R_12_: formyl	−0.067	R_2_&R_8_: Ac; R_23_: F	+0.119
R_3_: Ac; R_12_: formyl; R_23_: H	−0.057	R_3_: Ac; R_8_: formyl; R_23_: F	+0.110
R_3_: Ac; R_12_: formyl; R_23_: Et	−0.065	R_3_, R_8_&R_23_: Ac	+0.124
R_3_: Ac; R_12_: formyl; R_23_: Vi	−0.064	R_7_&R_23_: formyl;R_8_: Vi (“DVChl *bf*”)	+0.143
*R*_*3*_*: Ac; R*_*12*_: formyl *(“3Ac-Chl+”)*	*-0.059*		

Similar to the other Chl modifications listed in Tables 2 and 3, multiple formyl and acetyl residues seem to be beneficial to control $\Delta {E_{0 - T1}}$. However, in contrast to the singlet states, triplet state control is apparently often also facilitated by fluorine and hydrogen, though these occurrences might be simply due to other residues (formyl, acetyl) playing the main role. To test the positioning of the Chl $\Delta {E_{0 - T1}}$compared to the oxygen singlet energies, we propose two compounds: DVChl *bf* for higher $\Delta {E_{0 - T1}}$, which is structurally close to DVChl *b*, and, for lower energies, R3-formylated 3-acetyl Chl, “3Ac-Chl+”, named in analogy to “Chl *f+*” above. Note that 3Ac-Chl+ is a double modification (and thus likely easier to obtain than a triple modification), however with similarly shifted triplet energy as the set of triple modifications.

## Summary and conclusions

This contribution presents a series of new Chl compounds that, to the best of our knowledge, have not yet been realized, neither in the lab nor in vivo. The new Chls were predicted from scanning a complete set of Chl variants (within the limits of the chosen substitution sites and set, Fig. 1). by applying an ML-assisted screening of a closed chemical space of over 250,000 possible derivatives. Here, target properties (namely excitation energies and oscillator strengths) of selected optical excitations were predicted in (TD)-DFT quality, yet based on computationally much less expensive descriptors in SQC quality. Due to possibly faulty initial guess structures or general instability, only about 1% (ca. 3,000) of the possible structures were not structurally converged. Further, a special selection criterion was developed to choose which excited state to train against, since the numerical ordering of states and/or their character is not necessarily always preserved across individual Chl variants. While training performances for some states are thus not fully satisfactory, they are sufficient for the intended overview of the ChlVS.

This way, Chls were identified that likely exhibit improved features for the following roles:

(i) Red shifted, intense Q band absorbance, “Chl *df*” and “Chl *f+*”
(ii) Red shifted, intense B band absorbance, “Chl *bb*”, “Chl *bd*” and DVChl *b*
(iii) Adjusted T_1_ energies with minimal changes to Q_y_, “DVChl *bf*” and “3-Ac Chl*+*”

**Fig. 5:**
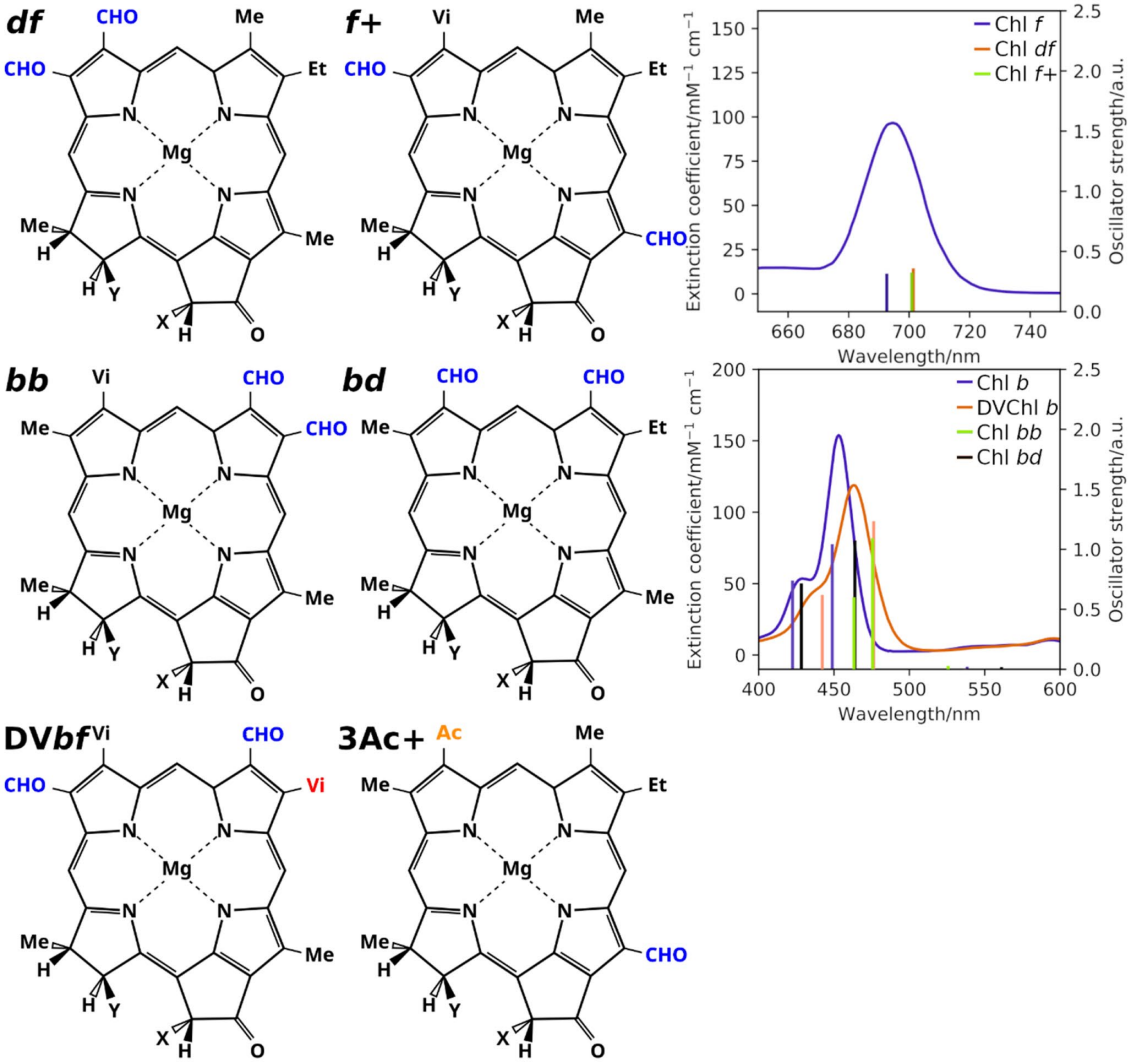
Chls with likely a good balance of experimental accessibility and interesting spectroscopic properties. Top row: Q band red shifted Chls. Middle row: B band red shifted Chls. Bottom row: triplet shifted Chls, while maintaining a Q_y_ excitation close to Chl *a*. For the bottom row chl properties, see table 4. The structural changes to Chl *a* are highlighted. For the sources of the experimental spectra, see figure 3; detailed values can be found in the SI, section S11, table S5. 3D representations of the compounds can be found in the SI, section S13

A summary of the predicted structures and spectra (if applicable) is shown in Fig. 5. None of the proposed structures contain fluorine, indicating that the introduction of this element will not be beneficial for the purposes intended here. The synthesis of the proposed compounds, however, is expected to be challenging (Liu et al. 2018; Woodward et al. 1960). Nevertheless, the predicted Chl variants may outperform the naturally occurring Chls, although this would likely require adjustment of the binding sites in the pigment-protein complexes (Lokstein et al. 2021). That being said, realization of these compounds in the lab could provide at least two different routes of future research: (i) Synthetic biology beyond the recombination approach, employing designed molecules that allow for better sunlight usage, photoprotection and thus, overall energy and material efficiency; and (ii) a direct test for the proposed roles of naturally occurring Chls.

While route (i) might be the most interesting one from an industrial perspective, route (ii) is crucial for the conceptual understanding of Chls and their role in photosynthesis. At the moment, the role assignment of photosynthetic pigment is often based on indirect evidence (such as spectroscopy on the wildtype or protein mutants). With the newly predicted Chl variants, it may be possible to retain the structural integrity of the protein yet still altering the pigment properties. This might allow for directly testing the proposed roles of Chls for photoprotection and energy transport, as can be done so far only for highly specific cases (Nürnberg et al. 2018). A major challenge now, however, will be to realize these newly predicted pigments via synthetic approaches.

## Electronic supplementary material

Below is the link to the electronic supplementary material.

Supplementary Material 1

## Data Availability

The supporting information data of this article is available as a Supplementary Information document: S1: Core structure used in this study. S2: Training settings of ArchOnML. S3: Learning curves for all models. S4: Distribution of training data. S5: Distribution of prediction data. S6: ML procedure, descriptors and ML model applied in this study. S7: Criterion for B band state selection in the ML scheme. S8: Comparison of ML data to conventional QC: TD-DFT (gas phase and acetone). S9: Comparison of ML data to conventional QC: DFT/MRCI. S10: Example QC inputs during the ML scheme. S11: Predicted values in Figures 3 and 5 of the main article. S12: Initial geometry generation procedure within ArchOnML. S13: 3D structures for candidates of interest. The code for ArchOnML can be found at https://github.com/archonml/archonml. The program version used was 1.0. The full ML models for use with the ArchOnML program are uploaded at the data repository of the Freie Universität Berlin, http://dx.doi.org/10.17169/refubium-47703.
