# Supplementary material for: New chlorophylls designed by theoretical spectroscopy and machine learning
Source: Photosynth Res. 2025 Dec 11;163(6):67. doi: 10.1007/s11120-025-01183-0 (PMC12698835; doi:10.1007/s11120-025-01183-0)
Supplement: Supplementary file 1 — Supplementary Material 1 [file 11120_2025_1183_MOESM1_ESM.pdf]

**New chlorophylls designed by theoretical spectroscopy and machine learning**

Fabian Weber,[a] Simon Petry,[a] Dennis J. Nürnberg[b,c] and Jan P. Götze[a,*]

[a]*Freie Universität Berlin, Fachbereich Biologie Chemie Pharmazie, Physikalische und Theoretische Chemie, Arnimallee 22, 14195 Berlin, Germany*

[b]*Freie Universität Berlin, Fachbereich Physik, Experimentelle Biophysik, Arnimallee 14, 14195 Berlin, Germany*

[c]*Freie Universität Berlin, Dahlem Centre of Plant Sciences, Albrecht-Thaer-Weg 6, 14195 Berlin, Germany*

**Corresponding author, email: jan.goetze@fu-berlin.de*

**Supporting information**

Table of contents

S1: Core structure used in this study

S2: Training settings of ArchOnML

S3: Learning curves for all models

S4: Distribution of training data

S5: Distribution of prediction data

S6: ML procedure, descriptors and ML model applied in this study

S7: Criterion for B band state selection in the ML scheme

S8: Comparison of ML data to conventional QC: TD-DFT (gas phase and acetone)

S9: Comparison of ML data to conventional QC: DFT/MRCI

S10: Example QC inputs during the ML scheme

S11: Predicted values in Figures 3 and 5 of the main article

S12: Initial geometry generation procedure within ArchOnML

S13: 3D structures for candidates of interest

S1: Core structure used in this study

Below, the core structure of the all-hydrogen substituted chlorophyll is given in xyz-format. Comments given at the side (i.e. ! x1) are relevant for the ArchOnML package.

```
      57

C     2.234005   -0.357089  -0.315353
C     -1.349956  -1.465035  -0.250820
C     -1.997212  -2.721898  -0.311001
C     -3.432475  -2.505858  -0.427705
C     -3.642984  -0.943202  -0.378785
C     -1.009123  -3.695588  -0.265646  !X3
C     0.226336   -2.955004  -0.177569
N     -0.033190  -1.589971  -0.170385
C     1.525058   -3.461812  -0.126212
C     2.711368   -2.722234  -0.081215
C     4.046428   -3.275111  -0.019263  !X2
C     4.911257   -2.204794  0.000658   !X1
C     4.090433   -1.018416  -0.056902
N     2.781756   -1.349407  -0.102713
C     4.582791   0.312590   -0.067226
C     3.854923   1.480119   -0.129933
C     4.409427   2.834223   -0.137054  !X6
C     3.345891   3.695628   -0.210968  !X5
C     2.144590   2.866634   -0.232626
N     2.480369   1.560415   -0.182628
C     0.832243   3.363234   -0.297188
C     -0.358889  2.647309   -0.326015
N     -0.491129  1.307700   -0.286080
C     -1.822403  0.950376   -0.343289
C     -2.703133  2.174195   -0.508125
C     -1.709760  3.343299   -0.372634  !X4
Mg    1.202054   -0.006661  -0.189726
H     -1.851991  4.394341   -1.469398  !Y4
C     -3.918742  2.255200   0.419834
C     -3.605422  2.097020   1.906295
C     -4.807309  1.672525   2.708410
O     -5.676548  0.935403   2.315711
H     3.354039   5.185768   -0.301558  !Y5
H     5.841000   3.131376   -0.101509  !Y6
H     4.387777   -4.733148  0.061594   !Y2
H     -1.175873  -5.177883  -0.311561  !Y3
O     -4.328972  -3.309711  -0.533100
C     -4.452885  -0.455022  -1.579909
O     -3.926413  0.011834   -2.553671
O     -5.782341  -0.529983  -1.523350
C     -6.499148  -1.057085  -0.408281
O     -4.803838  2.167784   3.949336
H     6.405496   -2.219602  0.068885   !Y1
H     5.666915   0.417194   -0.015978
H     0.733408   4.448033   -0.321626
H     1.626487   -4.548932  -0.127331
```

| | | | |
|---|---|---|---|
| H | -5.583138 | 1.805889 | 4.403665 |
| H | -3.176030 | 3.002191 | 2.355978 |
| H | -4.422827 | 3.220559 | 0.249299 |
| H | -4.207390 | -0.741871 | 0.545717 |
| H | -7.552826 | -1.059457 | -0.712413 |
| H | -6.177466 | -2.082907 | -0.191203 |
| H | -6.383258 | -0.414093 | 0.476854 |
| H | -1.850652 | 3.841875 | 0.602892 |
| H | -3.087311 | 2.141354 | -1.541419 |
| H | -4.656259 | 1.494165 | 0.137686 |
| H | -2.860455 | 1.294262 | 2.050497 |

S2: Training settings of ArchOnML

For determining the hyperparameters of the Kernel Ridge Regression (KRR) models, a grid-based scan is performed in a 5-fold cross-validation. To define the scanned grid regions, four parameters called `MinMod`, `MaxMod`, `Lambda_Bot` and `Lambda_Top` have to be given by the user. The first two determine how ArchOnML's dynamic grid set-up procedure modifies the $\sigma$ region, and the latter two give the lowest and highest values of the $\lambda$ grid in exponential ($10^N$) fashion. These parameters are currently determined on a trial-and-error basis for each trained property, using sparse grid resolutions at first, and then increasing grid resolution for the actual scan. For all models, the final resolution was chosen to be 32 × 32 grid-points. Below, the settings for all properties will be listed.

- $\Delta E(Q_1)$: `MinMod = 0.00001, MaxMod = 100, Lambda_Bot = -6, Lambda_Top = 1`
- $f(Q_1)$: `MinMod = 0.01, MaxMod = 50, Lambda_Bot = -4, Lambda_Top = 1`
- $\Delta E(Q_2)$: `MinMod = 0.001, MaxMod = 50, Lambda_Bot = -6, Lambda_Top = 1`
- $f(Q_2)$: `MinMod = 1, MaxMod = 100, Lambda_Bot = -6, Lambda_Top = -1`
- $\Delta E(B_1)$: `MinMod = 1, MaxMod = 100, Lambda_Bot = -7, Lambda_Top = -3`
- $f(B_1)$: `MinMod = 0.001, MaxMod = 20, Lambda_Bot = -5, Lambda_Top = 1`
- $\Delta E(B_2)$: `MinMod = 0.1, MaxMod = 100, Lambda_Bot = -5, Lambda_Top = -1`
- $f(B_2)$: `MinMod = 0.01, MaxMod = 20, Lambda_Bot = -5, Lambda_Top = 1`
- $E(T_1)$: `MinMod = 0.00001, MaxMod = 100, Lambda_Bot = -6, Lambda_Top = 1`

$Q_1$ was always assigned to be the $S_1/Q_y$ state, and $Q_2$ to be $S_2/Q_x$. The assignment of the B states was more involved, see section S6.

S3: Learning curves for all models

On the following pages, the learning curves for all nine trained properties will be presented (S3a-i). As described in the main text, five individual learning curves were generated for each property to check the robustness of each model. Robustness means that for a different random training-testing-split, the overall performance does not change. Note that splitting off the test data was performed before stratification during training. This way, a completely random set of unknown molecules is used for checking performances, which reflects a more realistic situation.

The five individual curves will show both training and testing results for the coefficient of determination $r^2$ as well as the mean absolute error (MAE) at different amounts of training data during cross-validation. After these, the mean curves for $r^2$ are given, displaying both the best and worst individual results at each percentage – as well as the mean of all five curves for the training and testing, respectively.

S3a: Learning curves for $\Delta E(Q_1)$

## Curve 1

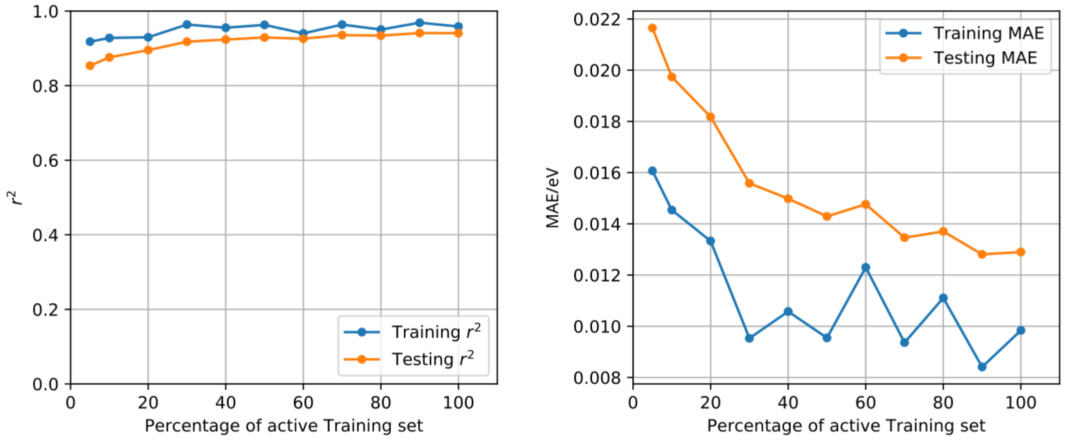


## Curve 2

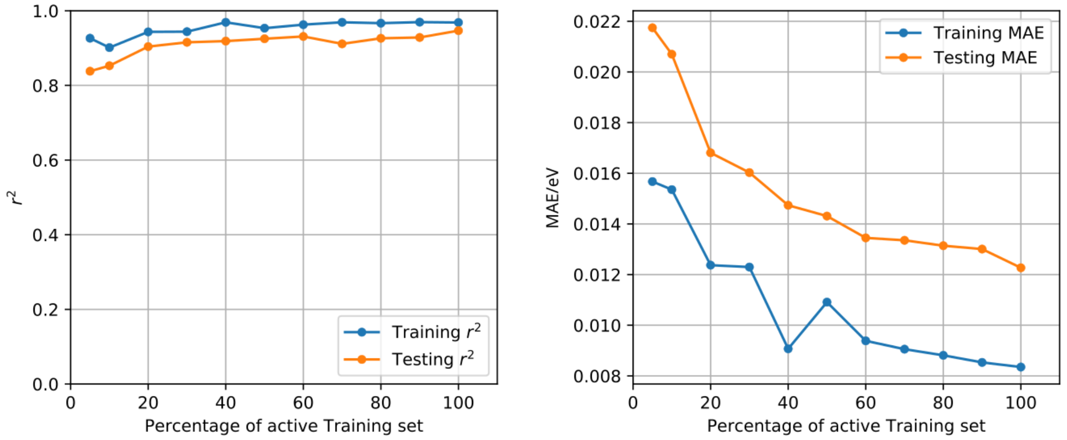


## Curve 3

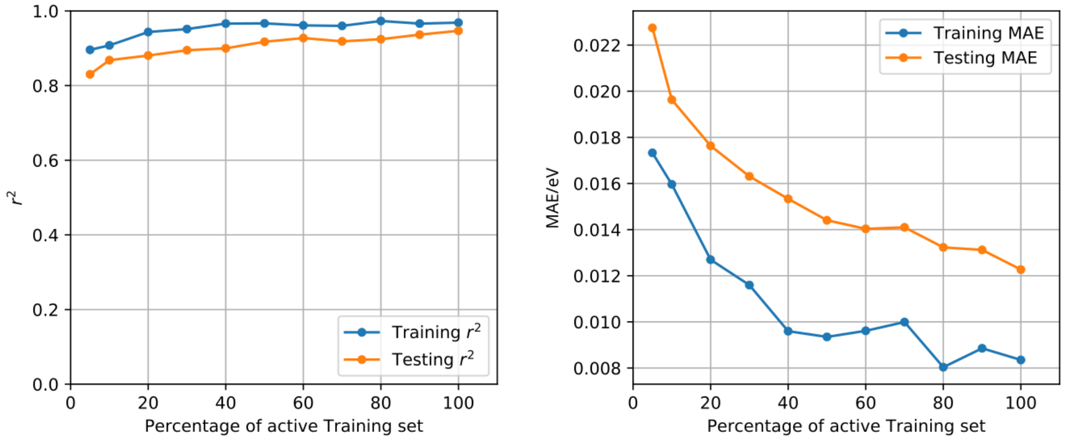

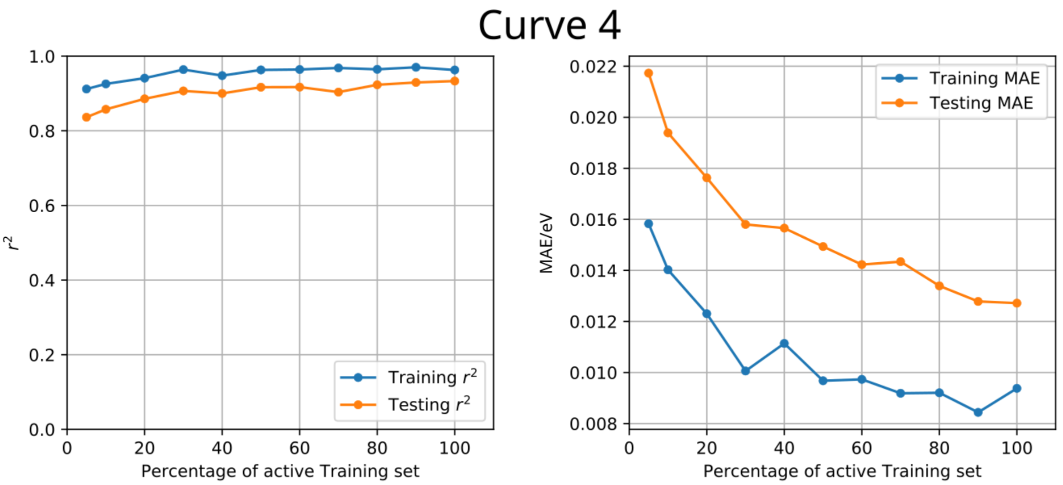
Curve 4
$r^2$
Training $r^2$
Testing $r^2$
Percentage of active Training set
MAE/eV
Training MAE
Testing MAE
Percentage of active Training set

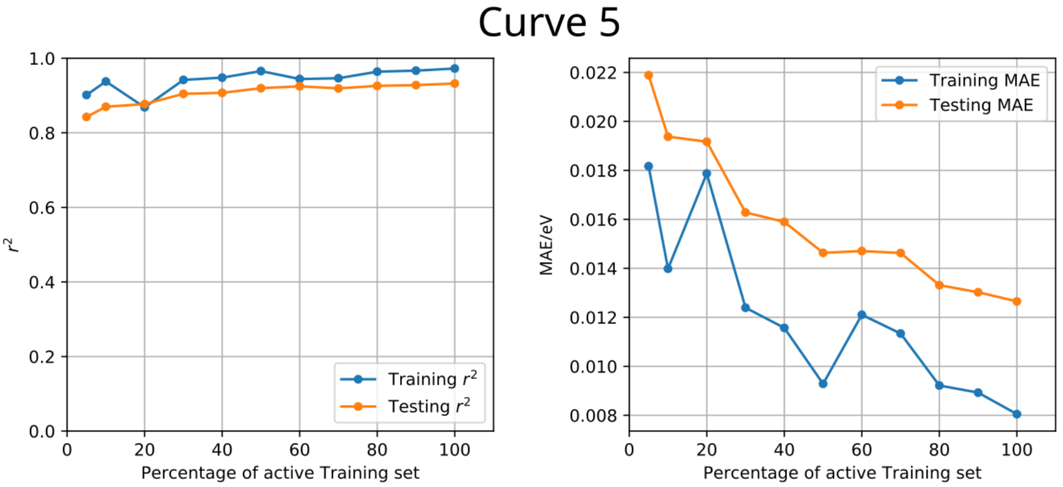
Curve 5
$r^2$
Training $r^2$
Testing $r^2$
Percentage of active Training set
MAE/eV
Training MAE
Testing MAE
Percentage of active Training set

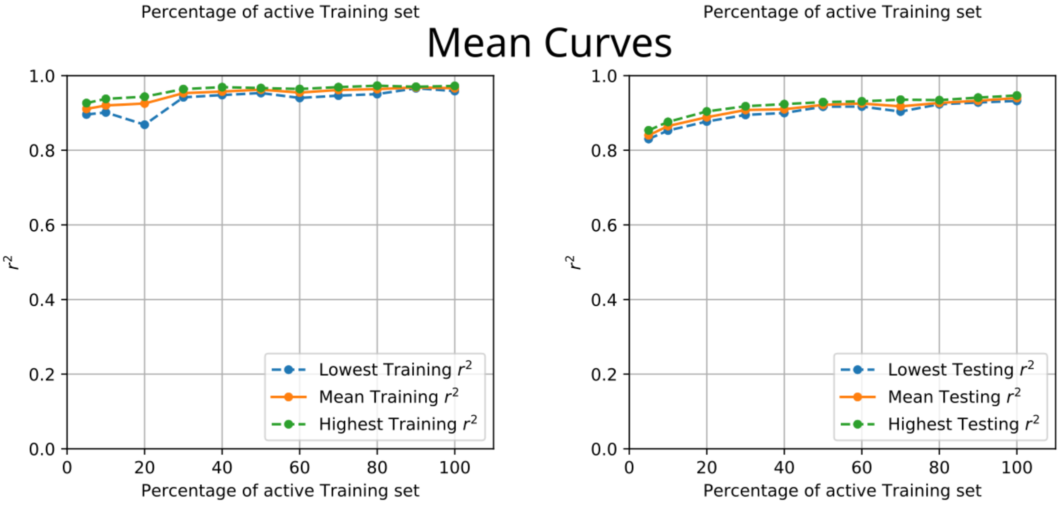
Mean Curves
$r^2$
Lowest Training $r^2$
Mean Training $r^2$
Highest Training $r^2$
Percentage of active Training set
$r^2$
Lowest Testing $r^2$
Mean Testing $r^2$
Highest Testing $r^2$
Percentage of active Training set

S3b: Learning curves for $f(Q_1)$

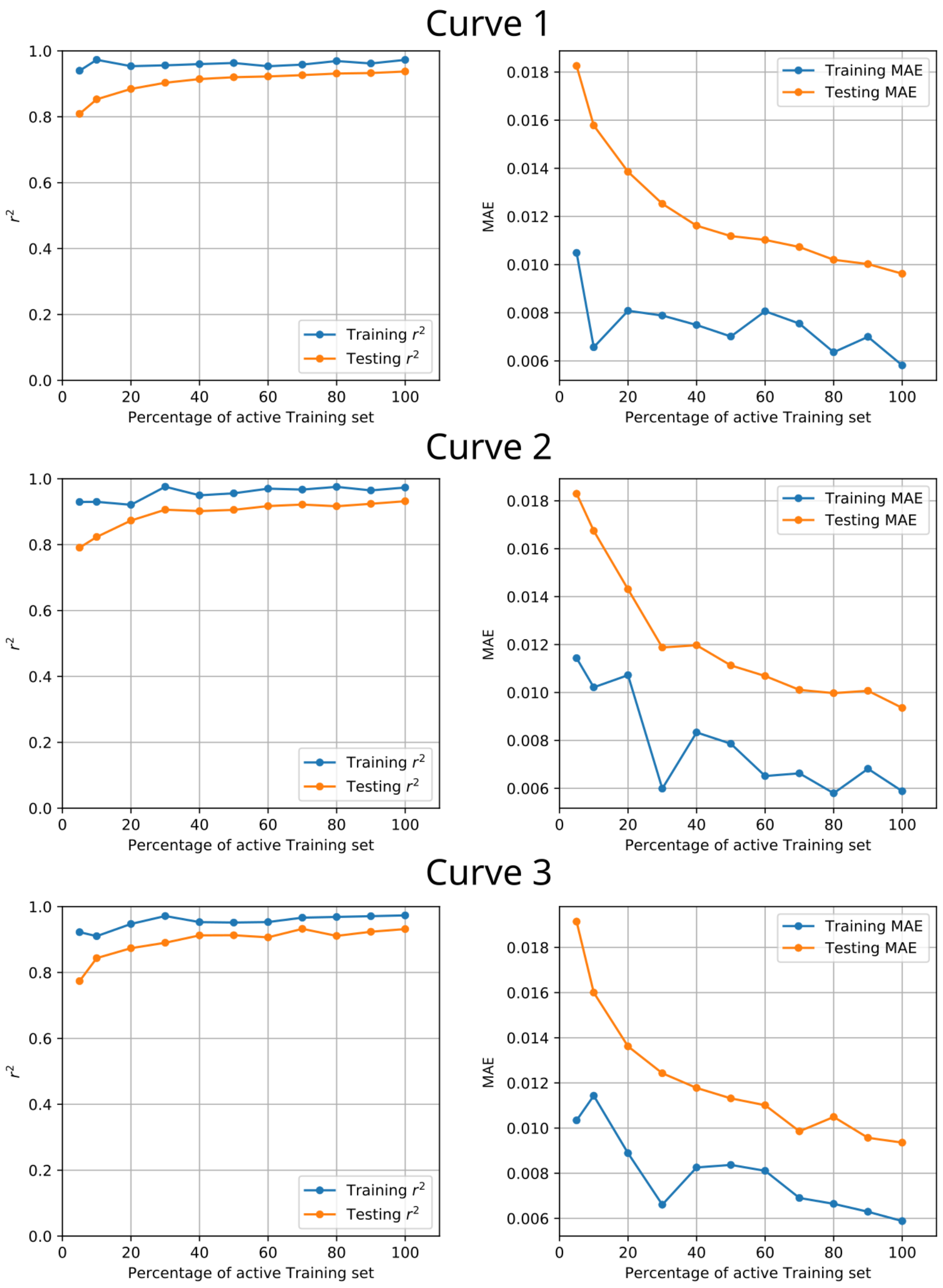

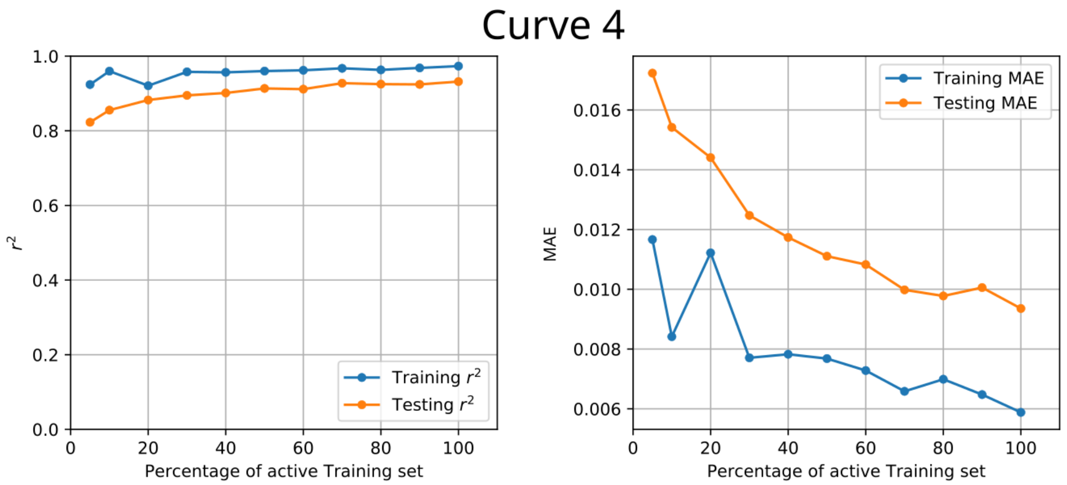
Curve 4
$r^2$
Percentage of active Training set
Training $r^2$
Testing $r^2$
MAE
Percentage of active Training set
Training MAE
Testing MAE

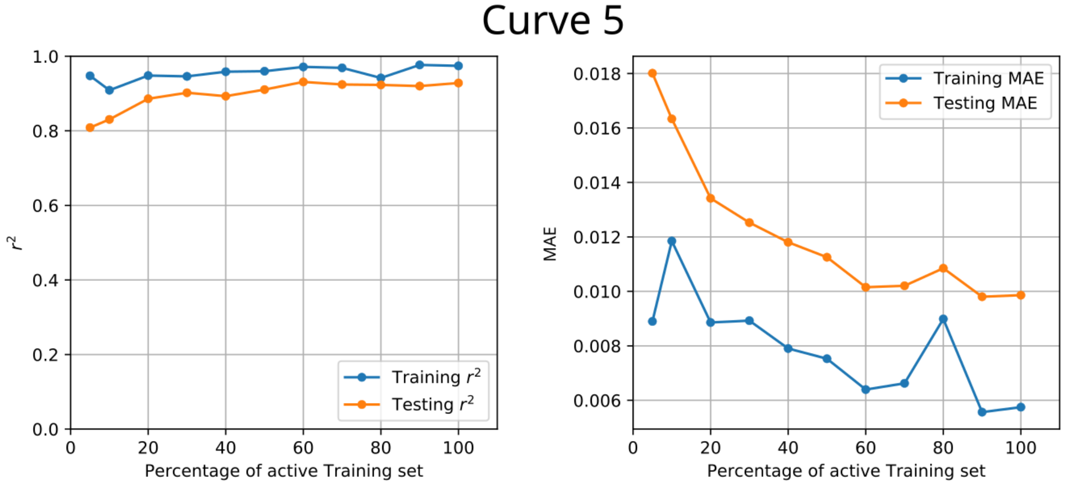
Curve 5
$r^2$
Percentage of active Training set
Training $r^2$
Testing $r^2$
MAE
Percentage of active Training set
Training MAE
Testing MAE

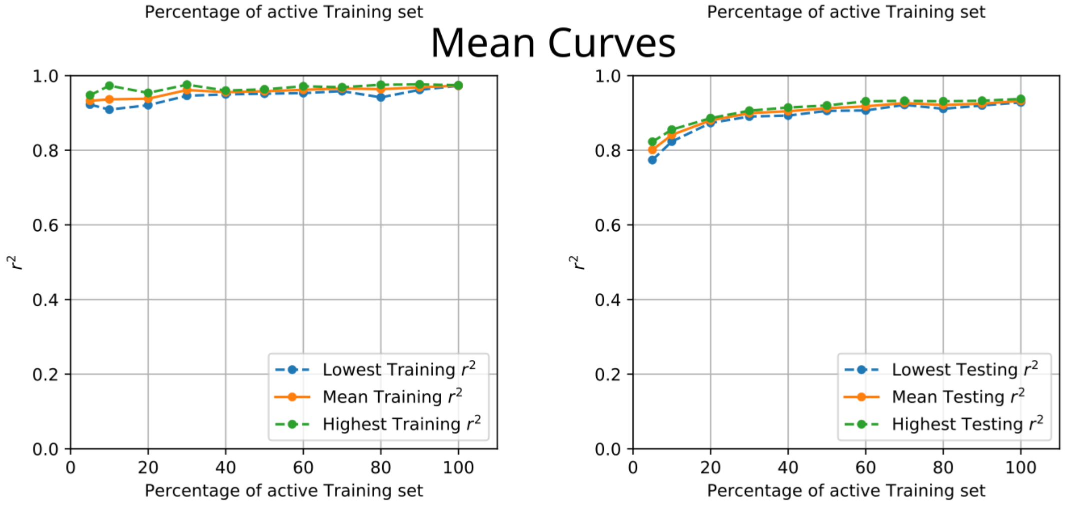
Mean Curves
$r^2$
Percentage of active Training set
Lowest Training $r^2$
Mean Training $r^2$
Highest Training $r^2$
$r^2$
Percentage of active Training set
Lowest Testing $r^2$
Mean Testing $r^2$
Highest Testing $r^2$

S3c: Learning curves for $\Delta E(Q_2)$

# Curve 1

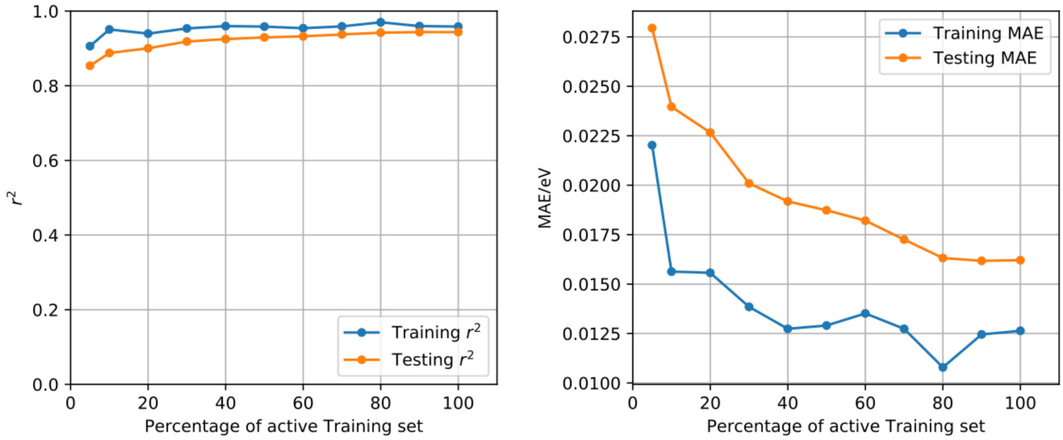


# Curve 2

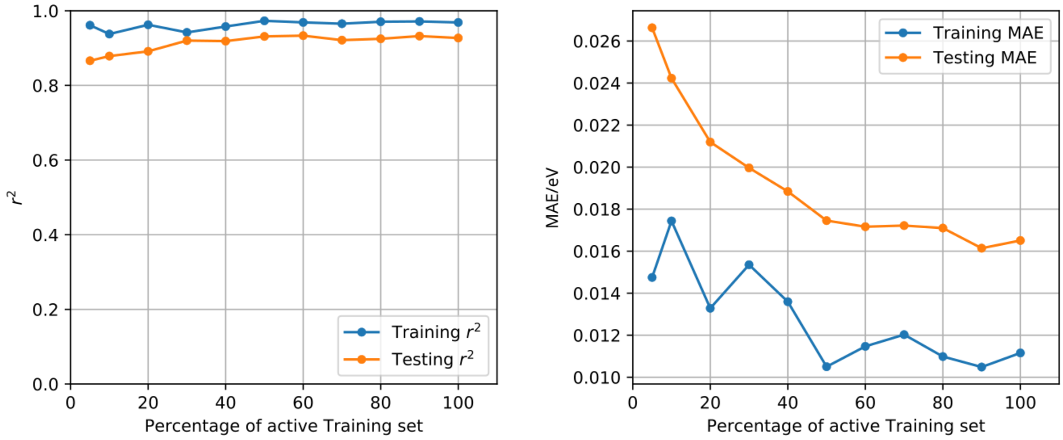


# Curve 3

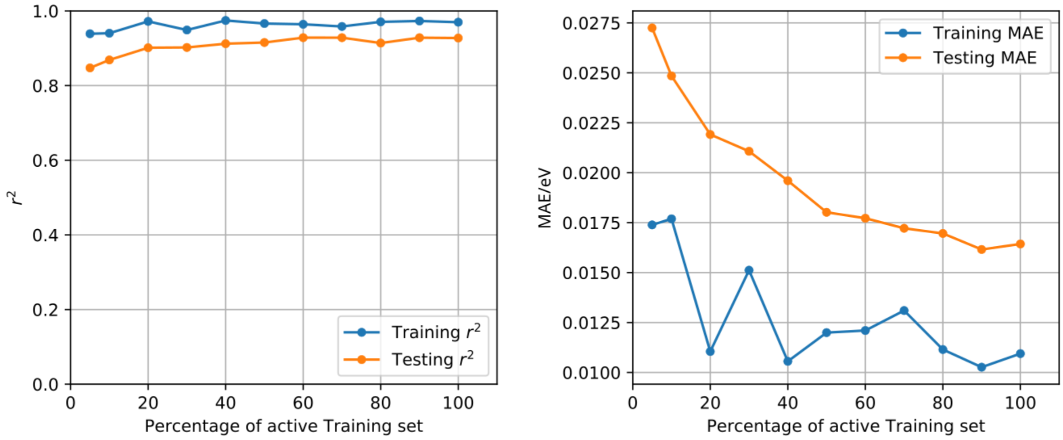

## Curve 4

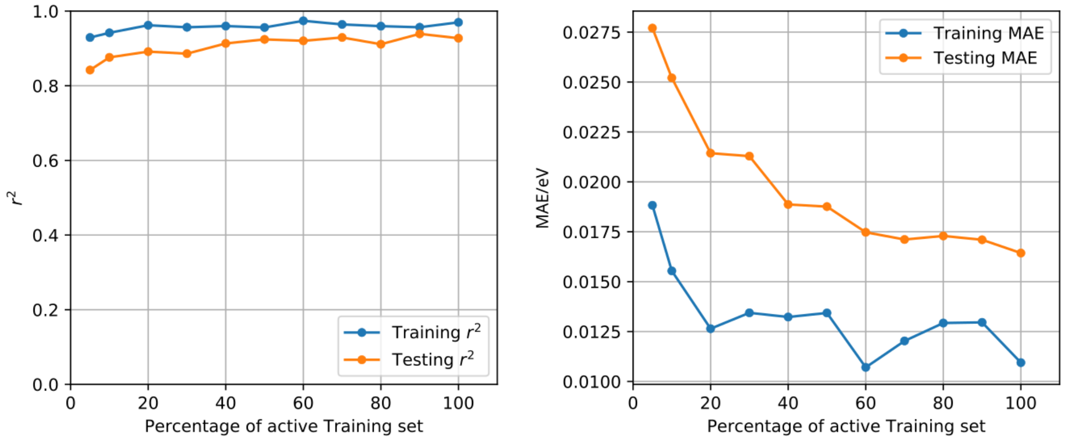


## Curve 5

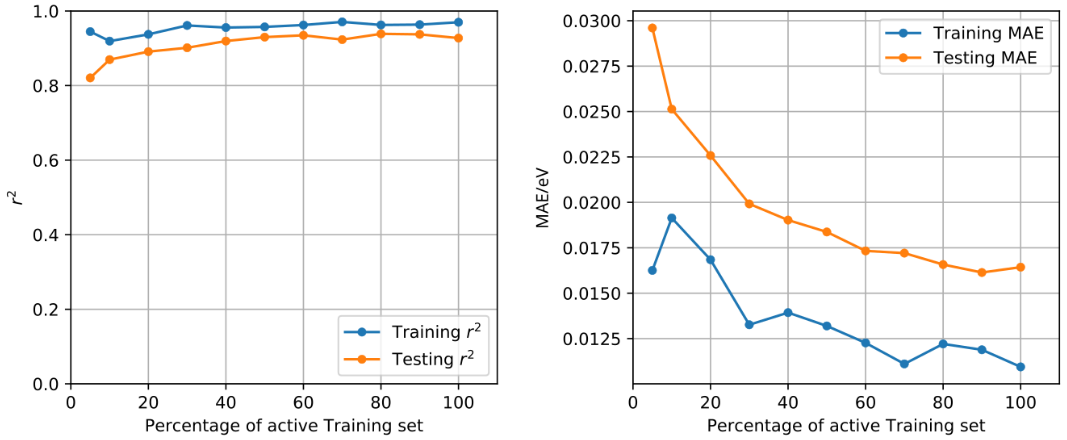


## Mean Curves

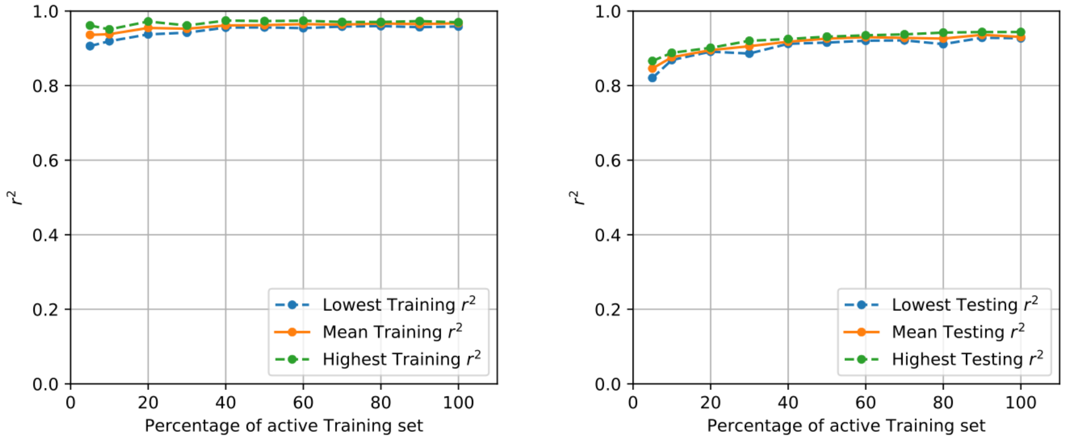

S3d: Learning curves for $f(Q_2)$

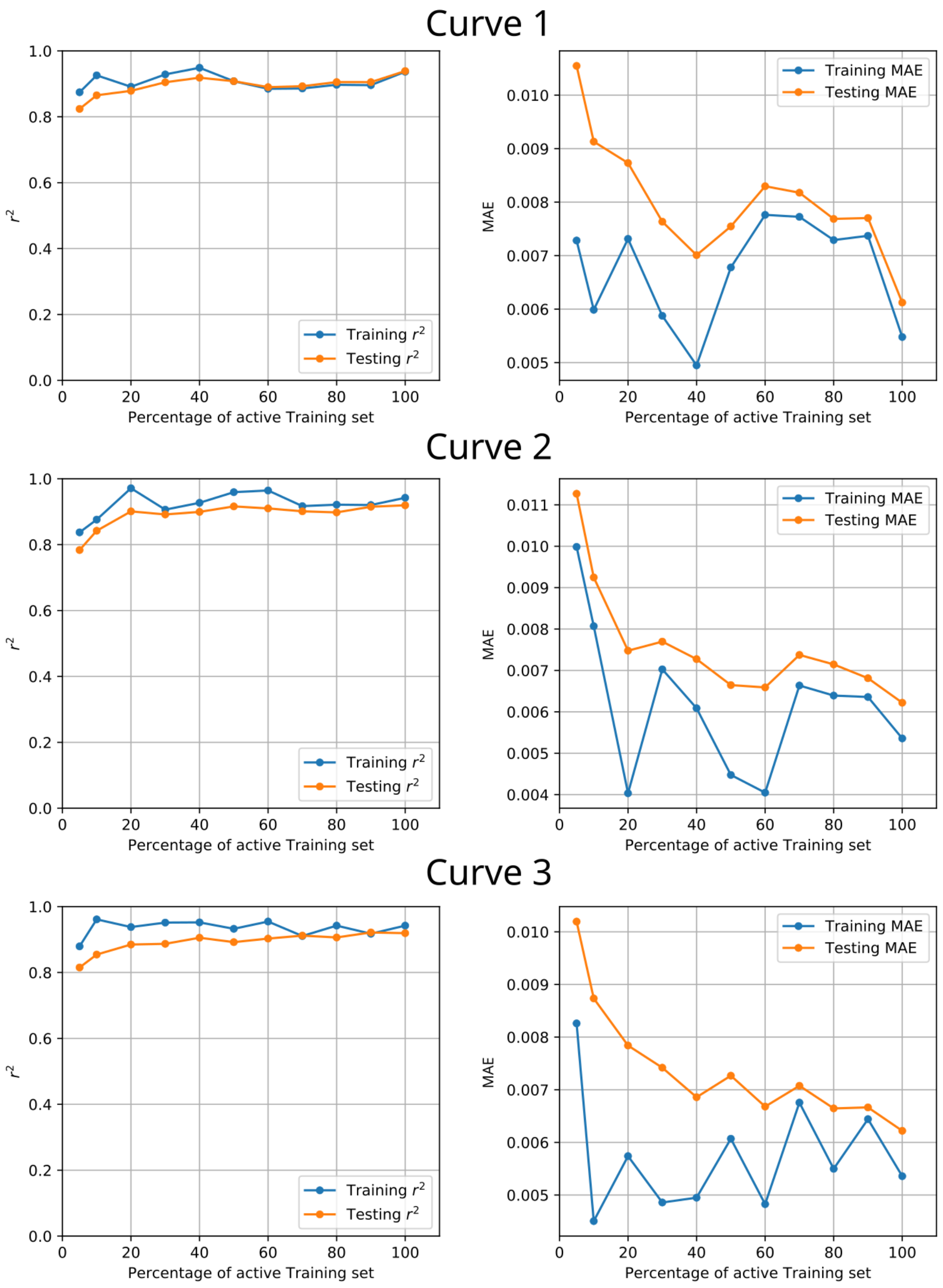

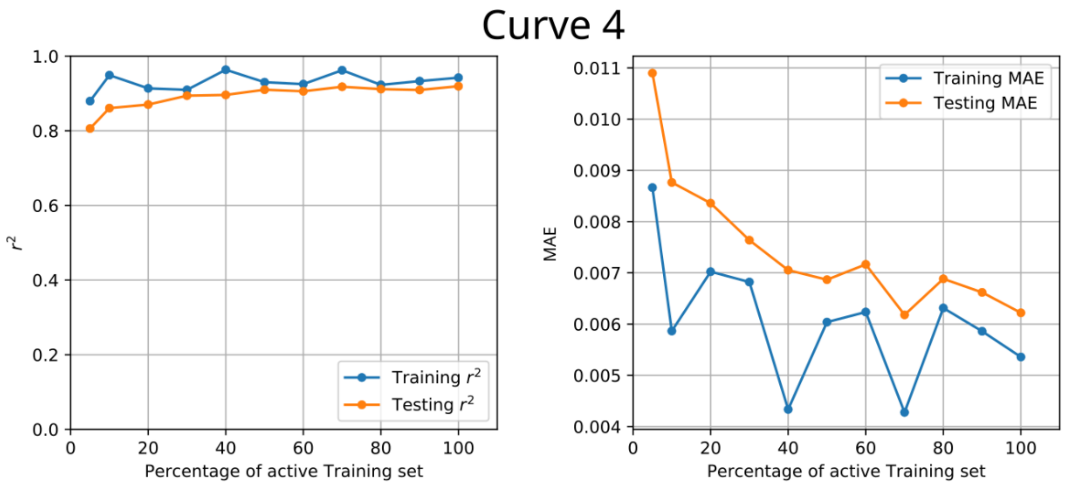
Curve 4
r²
MAE
Training $r^2$
Testing $r^2$
Training MAE
Testing MAE
Percentage of active Training set
Percentage of active Training set

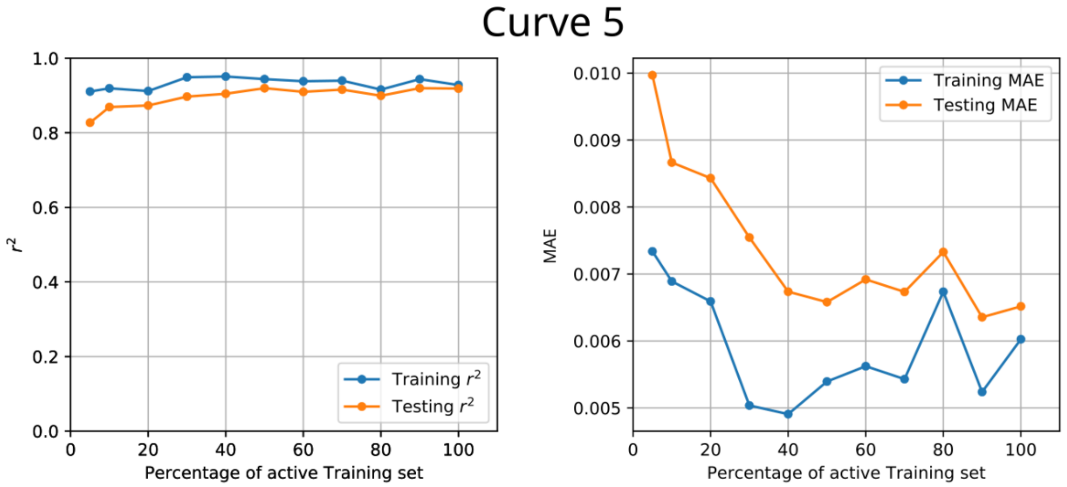
Curve 5
r²
MAE
Training $r^2$
Testing $r^2$
Training MAE
Testing MAE
Percentage of active Training set
Percentage of active Training set

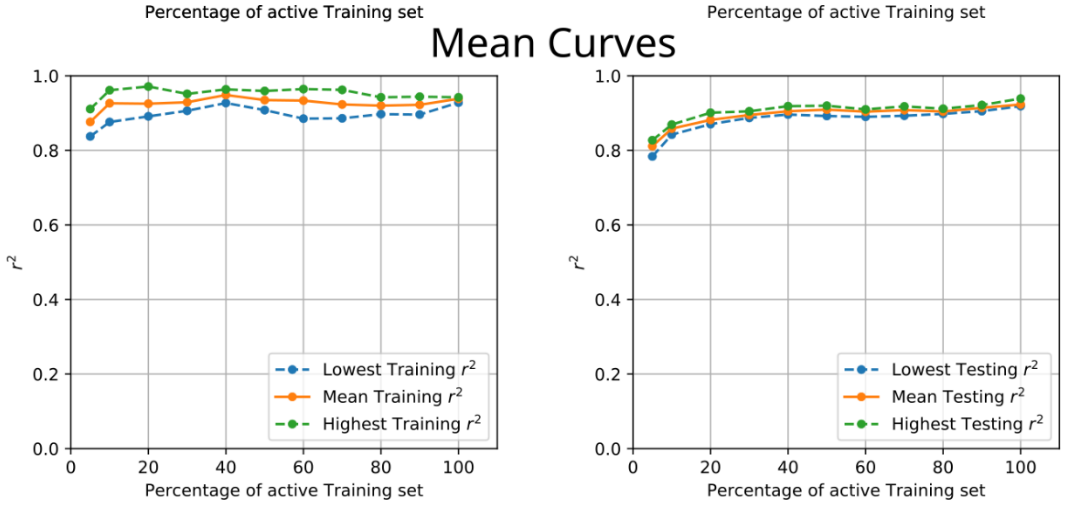
Mean Curves
r²
r²
Lowest Training $r^2$
Mean Training $r^2$
Highest Training $r^2$
Lowest Testing $r^2$
Mean Testing $r^2$
Highest Testing $r^2$
Percentage of active Training set
Percentage of active Training set

S3e: Learning curves for $\Delta E(B_1)$

Curve 1

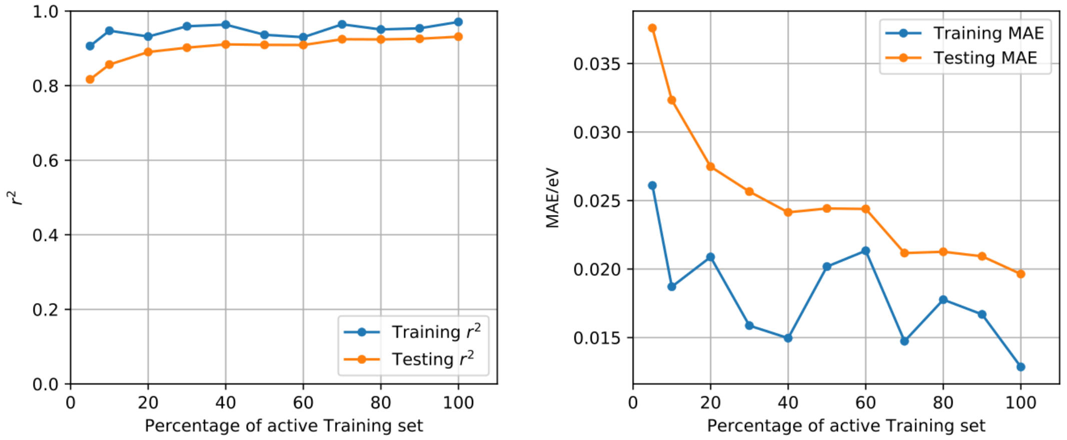


Curve 2

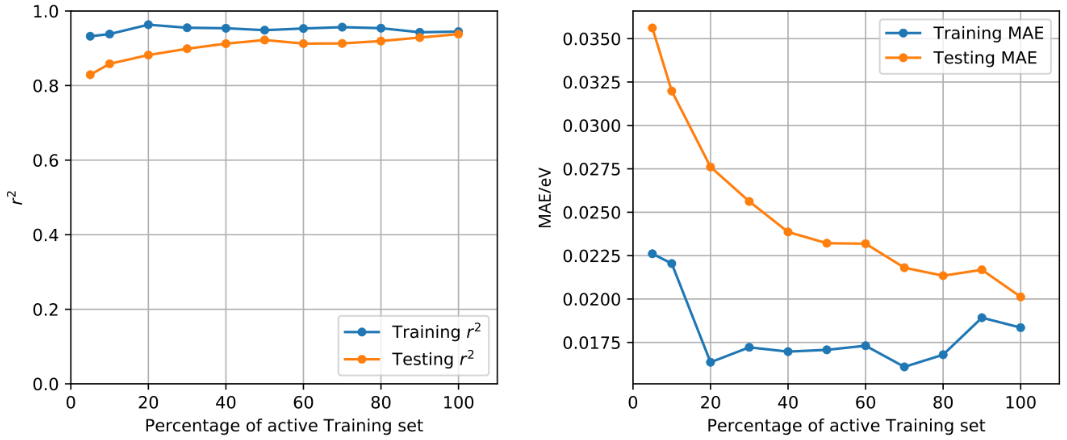


Curve 3

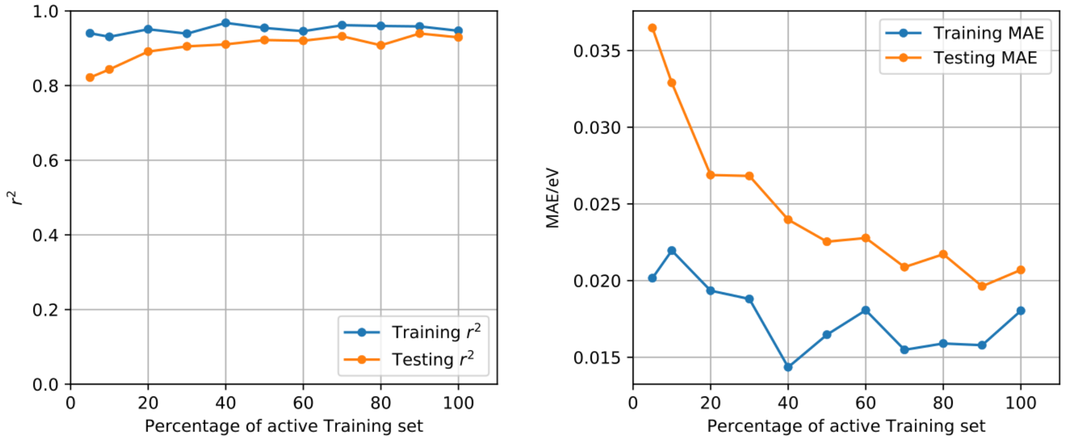

## Curve 4

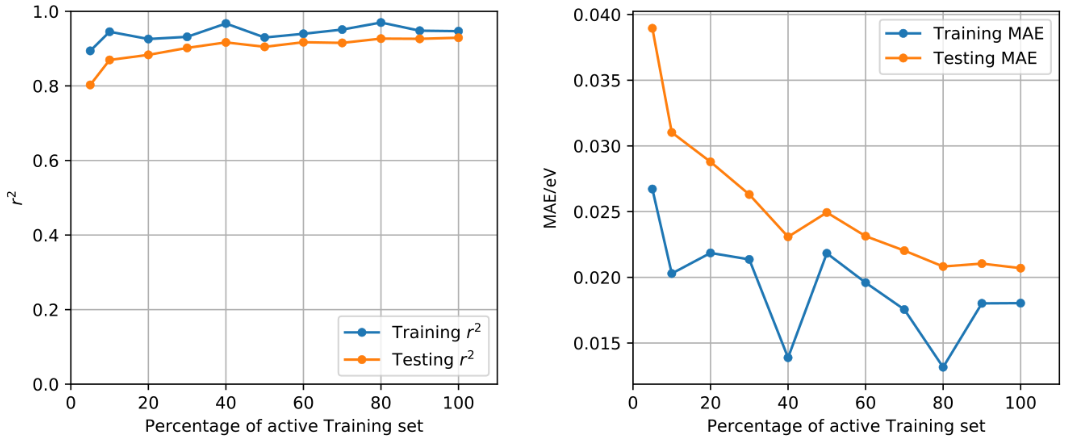


## Curve 5

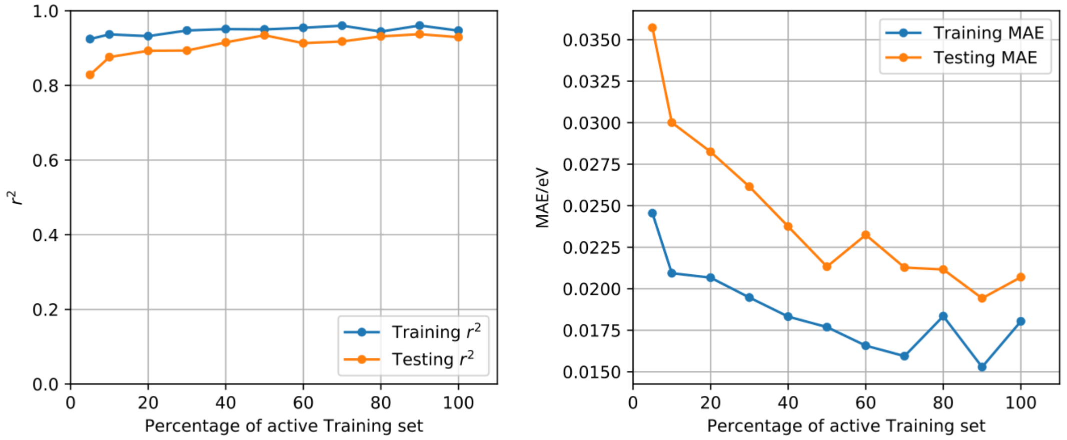


## Mean Curves

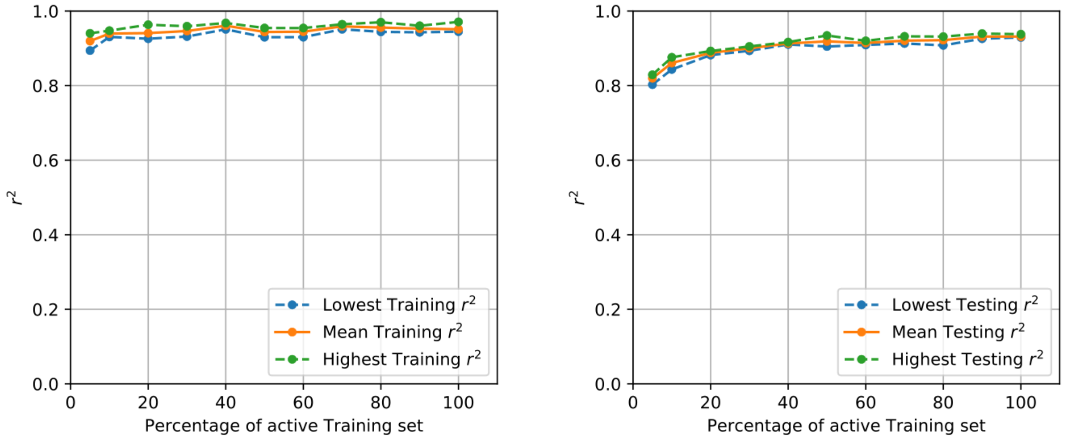

S3f: Learning curves for $f(B_1)$

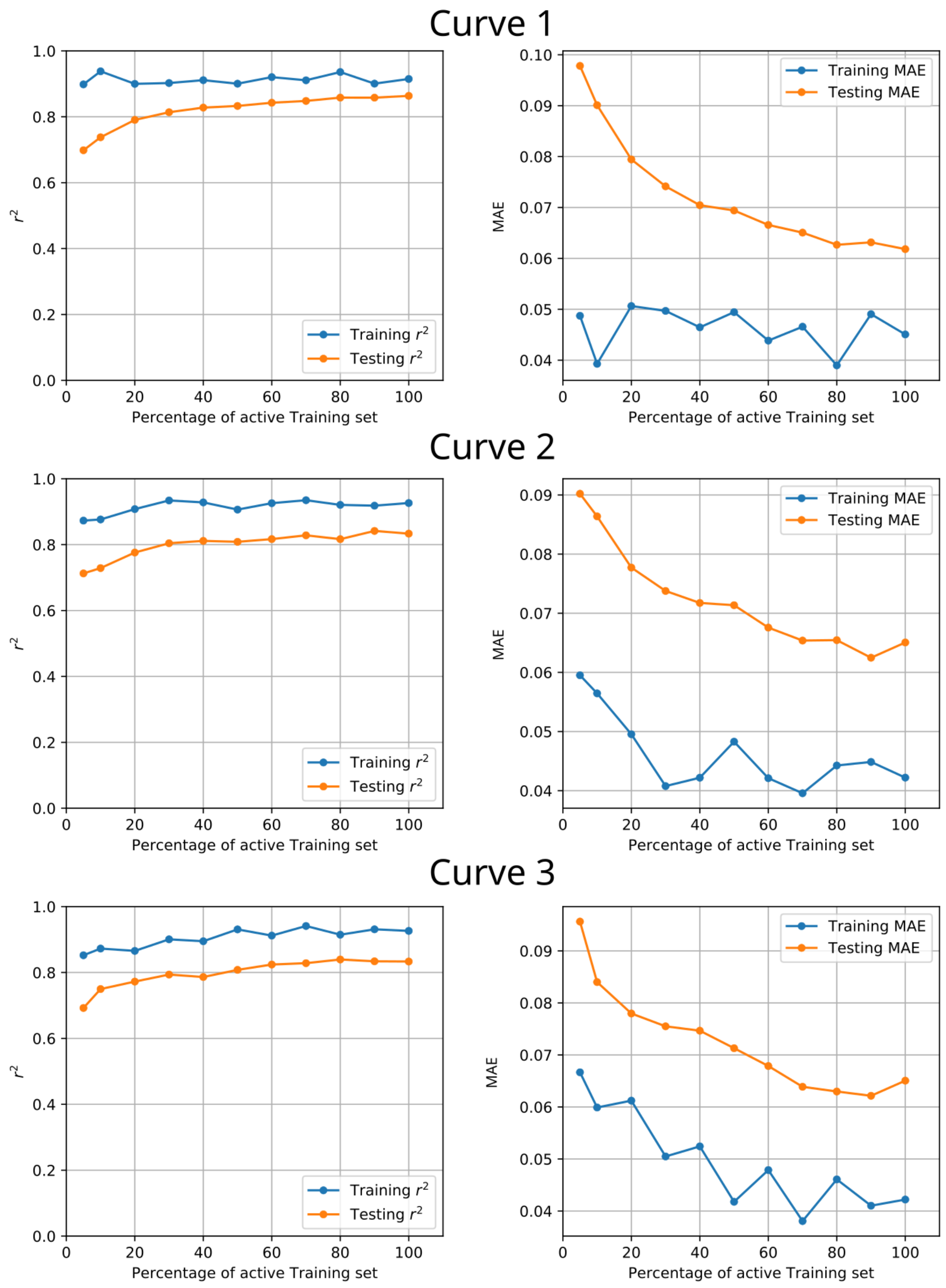

# Curve 4

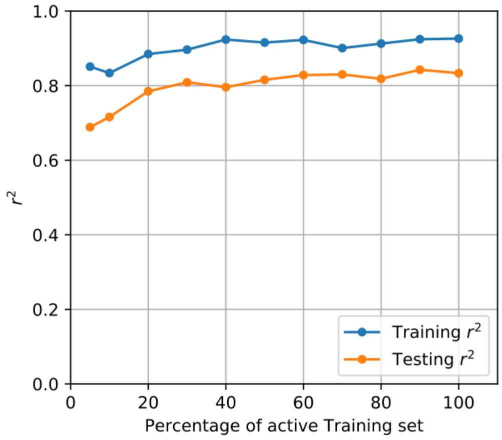


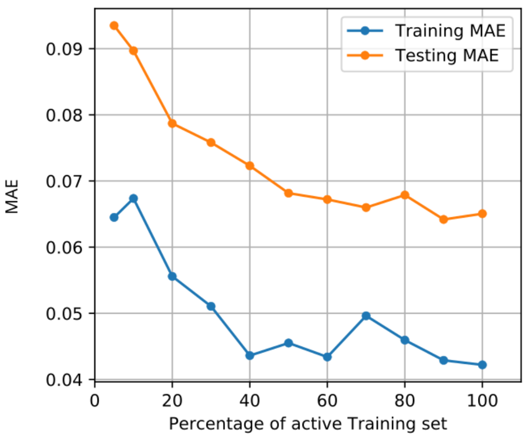


# Curve 5

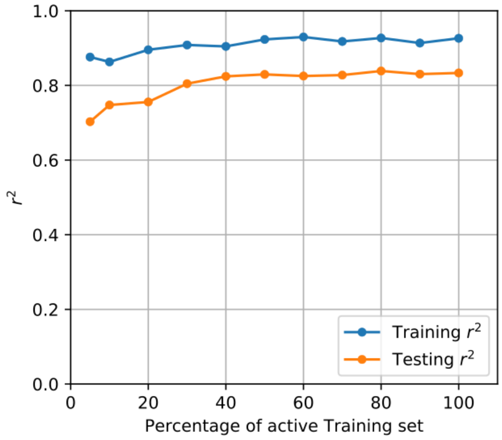


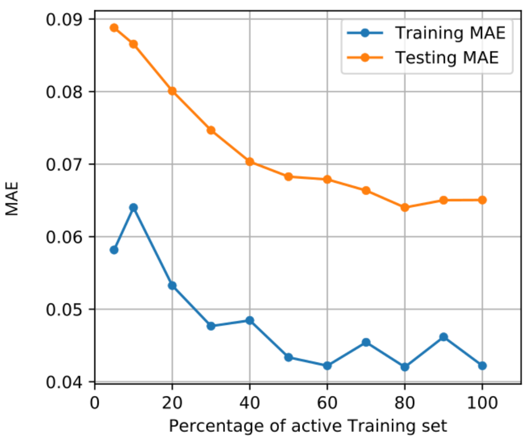


# Mean Curves

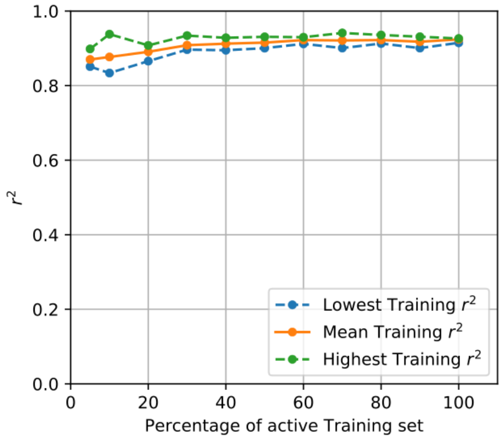


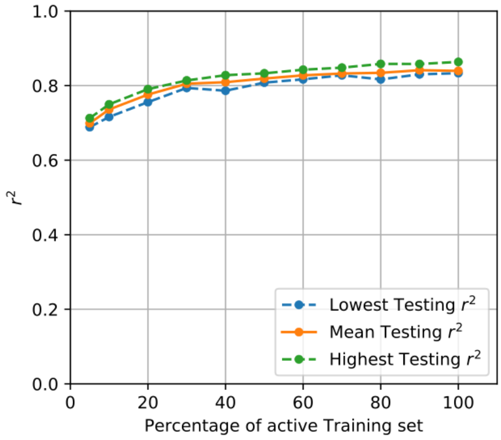

S3g: Learning curves for $\Delta E(B_2)$

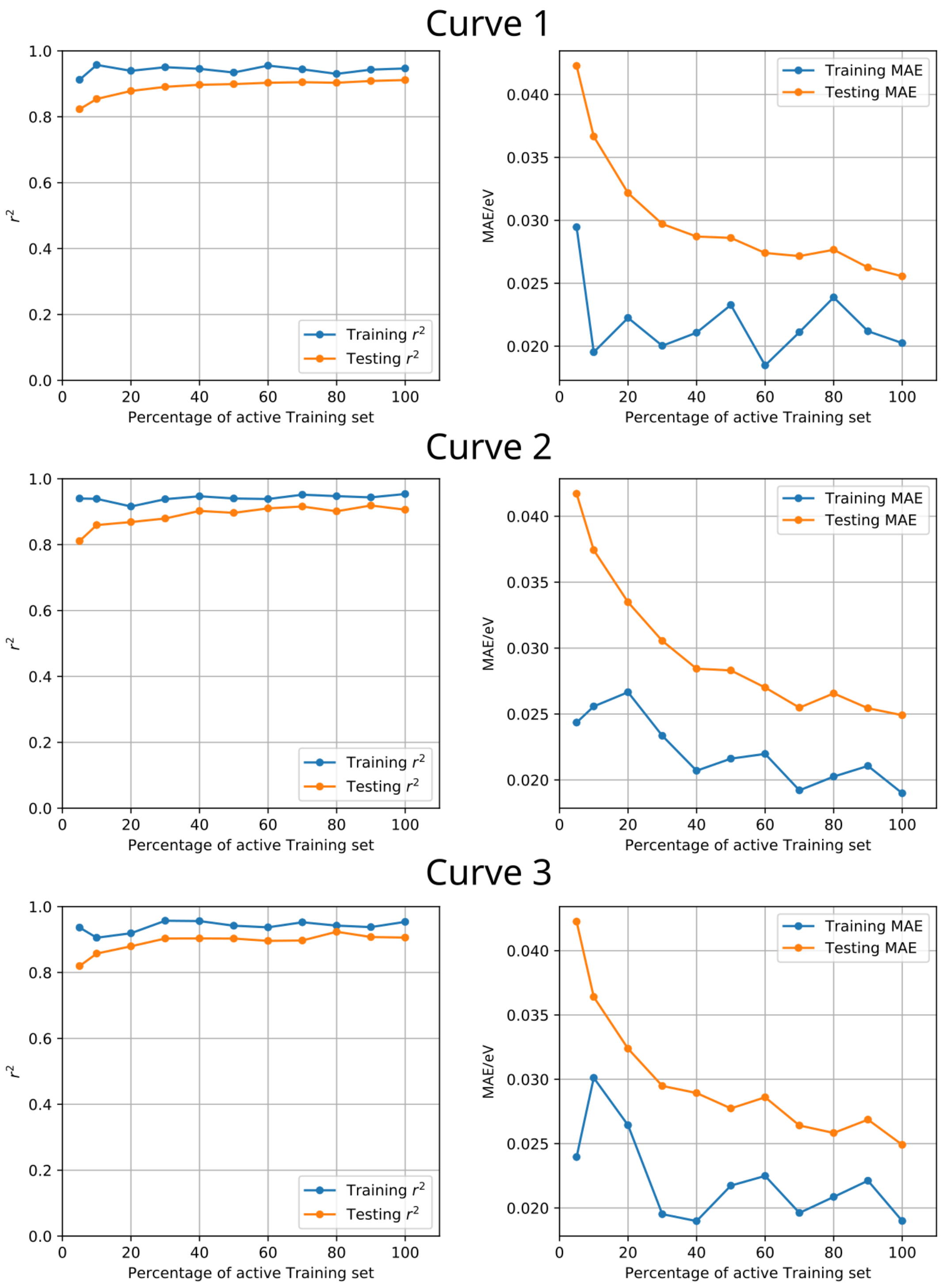

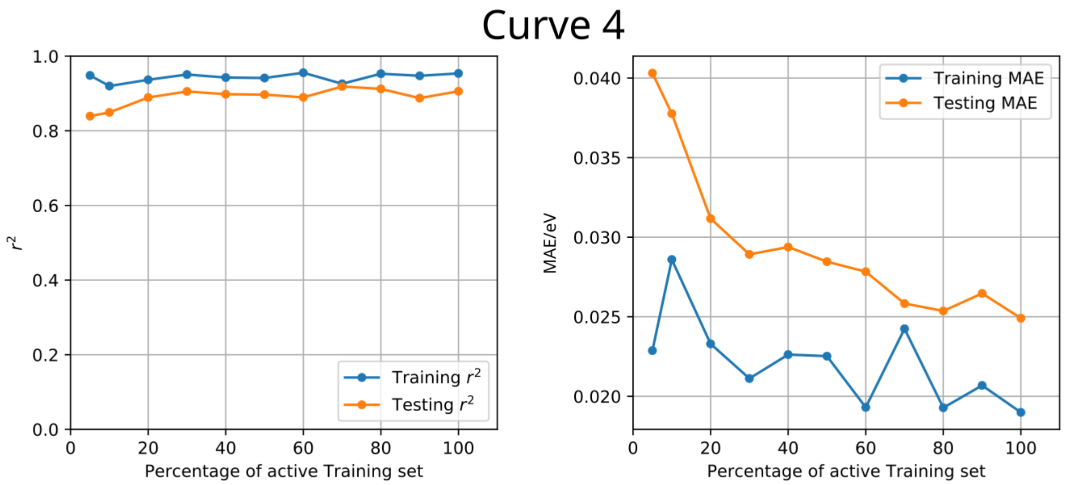
Curve 4
Training $r^2$
Testing $r^2$
$r^2$
Percentage of active Training set
Training MAE
Testing MAE
MAE/eV
Percentage of active Training set

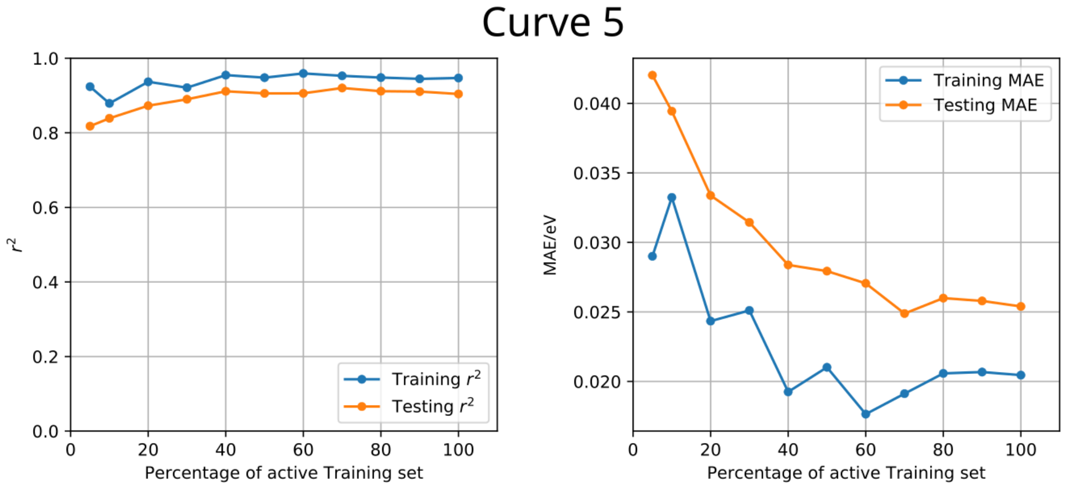
Curve 5
Training $r^2$
Testing $r^2$
$r^2$
Percentage of active Training set
Training MAE
Testing MAE
MAE/eV
Percentage of active Training set

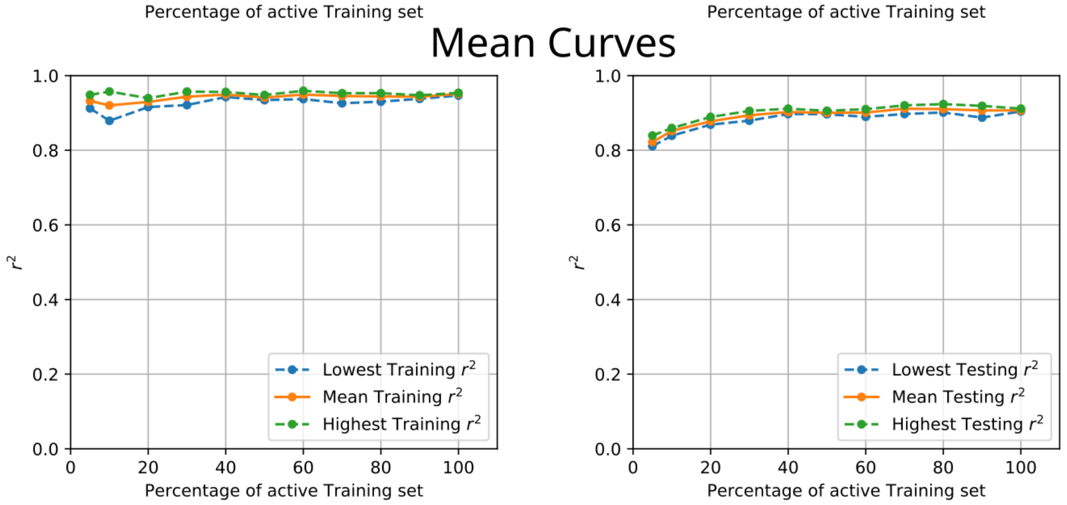
Mean Curves
Lowest Training $r^2$
Mean Training $r^2$
Highest Training $r^2$
$r^2$
Percentage of active Training set
Lowest Testing $r^2$
Mean Testing $r^2$
Highest Testing $r^2$
$r^2$
Percentage of active Training set

S3h: Learning curves for $f(B_2)$

## Curve 1

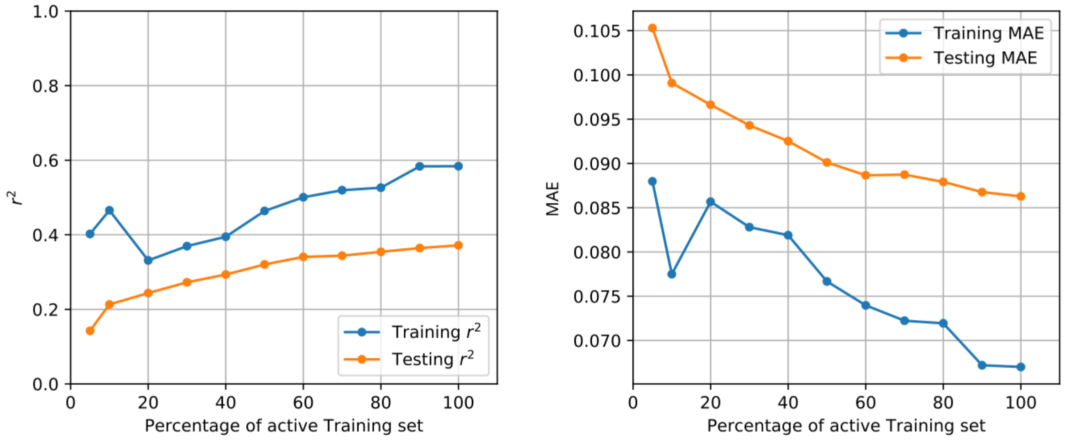


## Curve 2

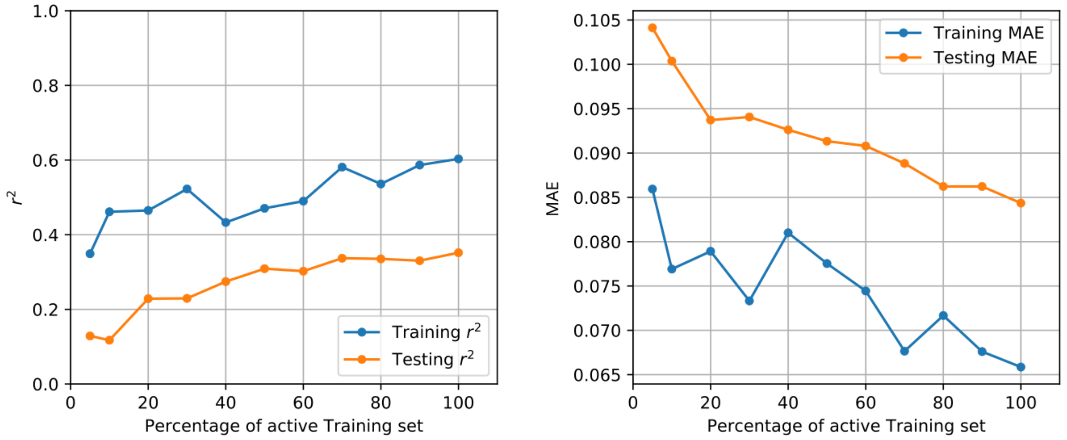


## Curve 3

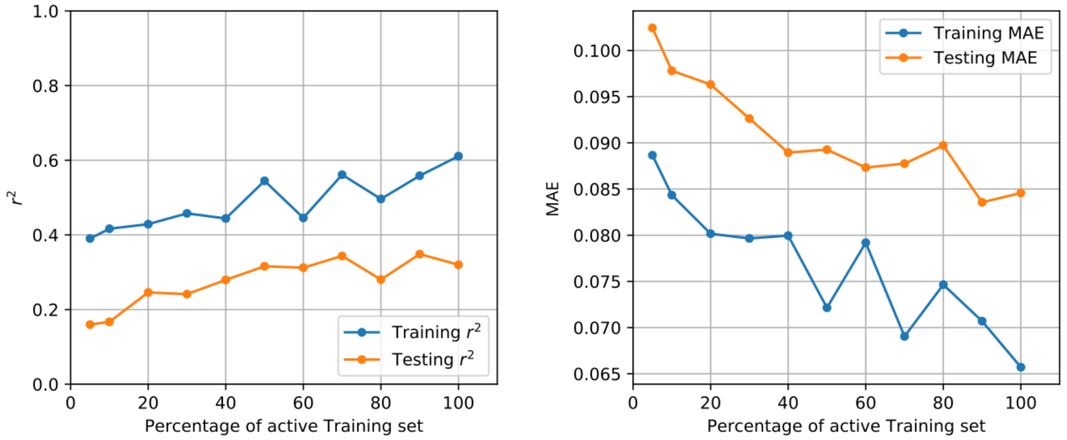

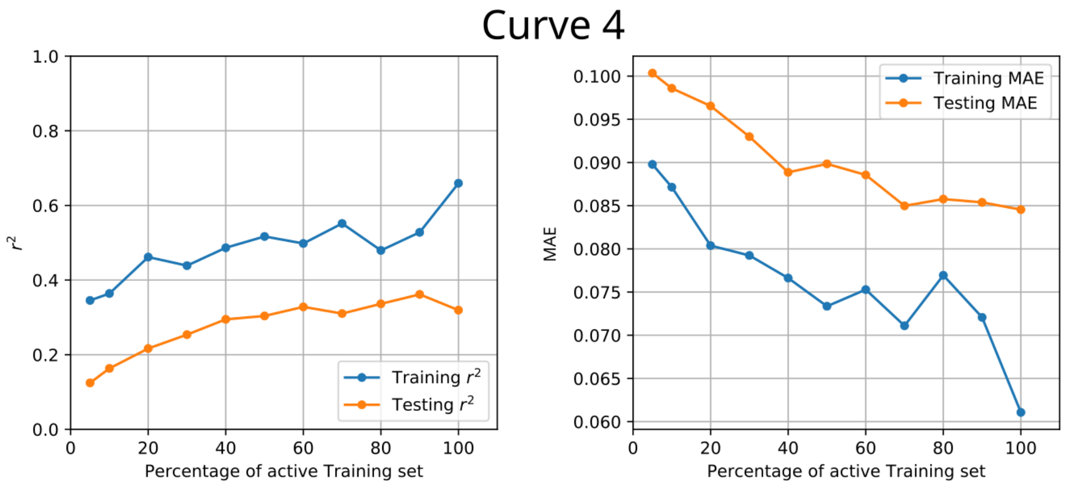
Curve 4
r²
Training r²
Testing r²
MAE
Training MAE
Testing MAE
Percentage of active Training set

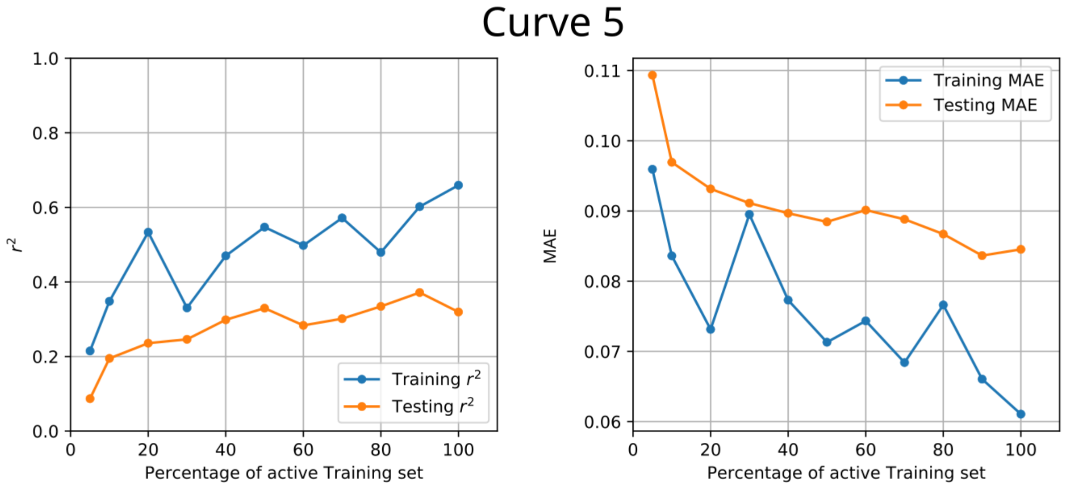
Curve 5
r²
Training r²
Testing r²
MAE
Training MAE
Testing MAE
Percentage of active Training set

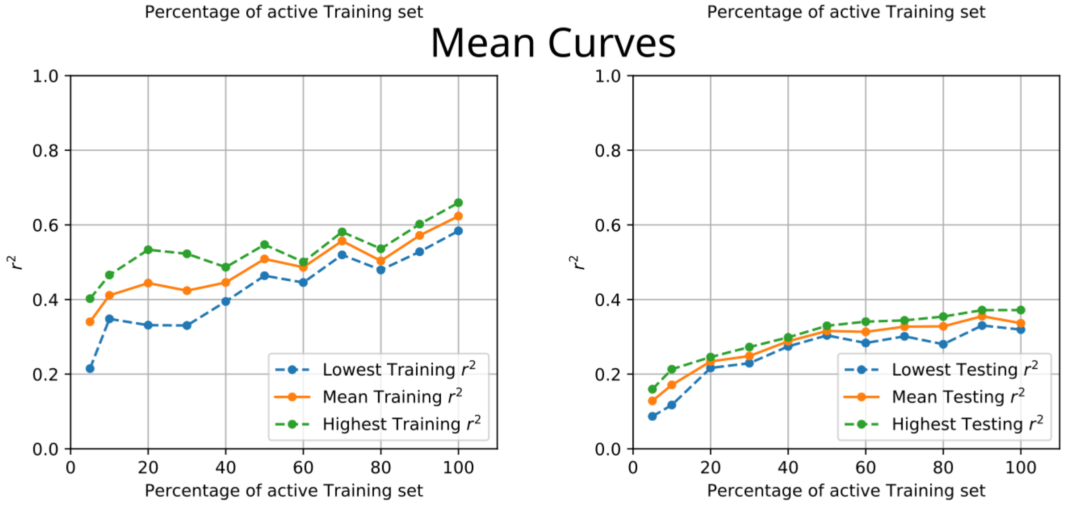
Mean Curves
r²
Lowest Training r²
Mean Training r²
Highest Training r²
Lowest Testing r²
Mean Testing r²
Highest Testing r²
Percentage of active Training set

S3i: Learning curves for $E(T_1)$

Curve 1

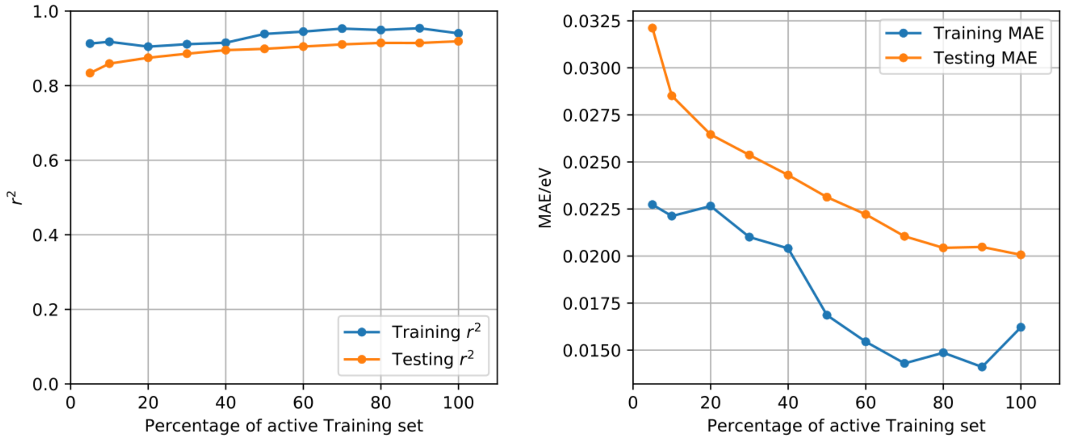


Curve 2

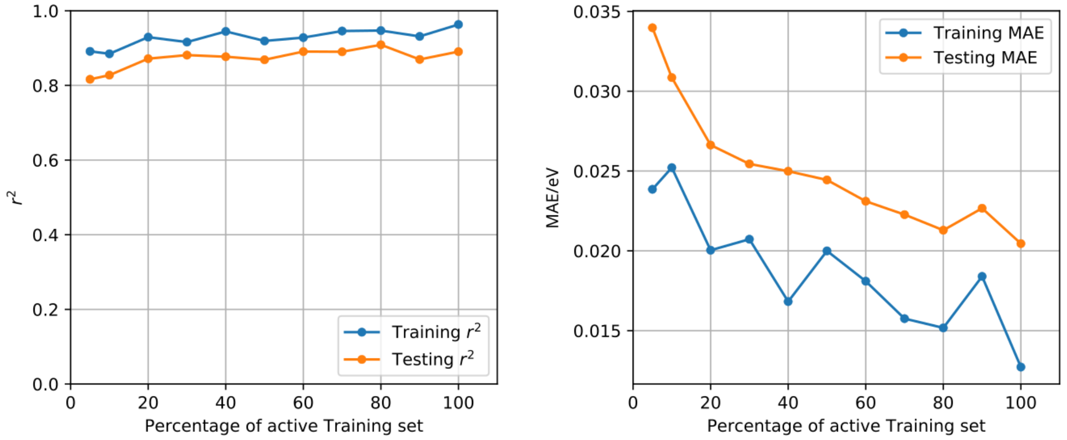


Curve 3

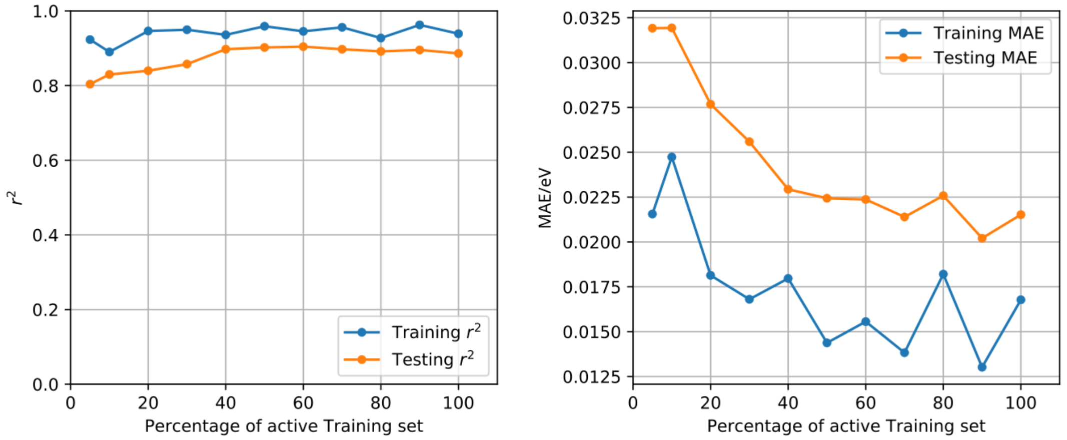

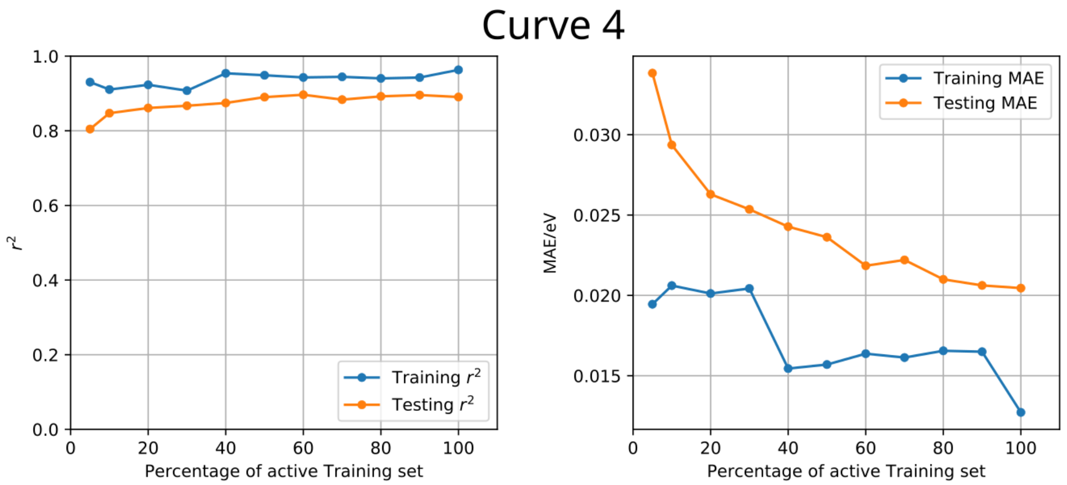
Curve 4
r²
Percentage of active Training set
Training $r^2$
Testing $r^2$
MAE/eV
Training MAE
Testing MAE

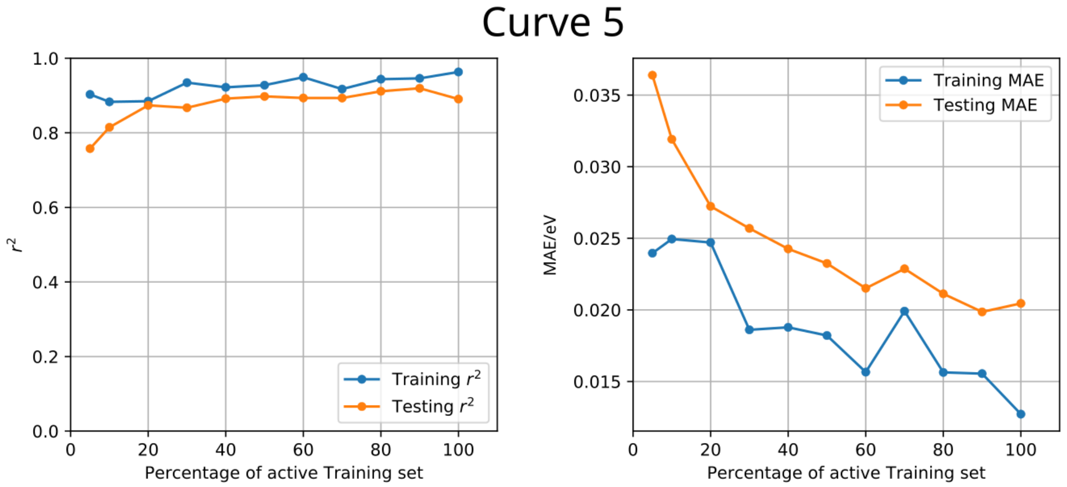
Curve 5
r²
Percentage of active Training set
Training $r^2$
Testing $r^2$
MAE/eV
Training MAE
Testing MAE

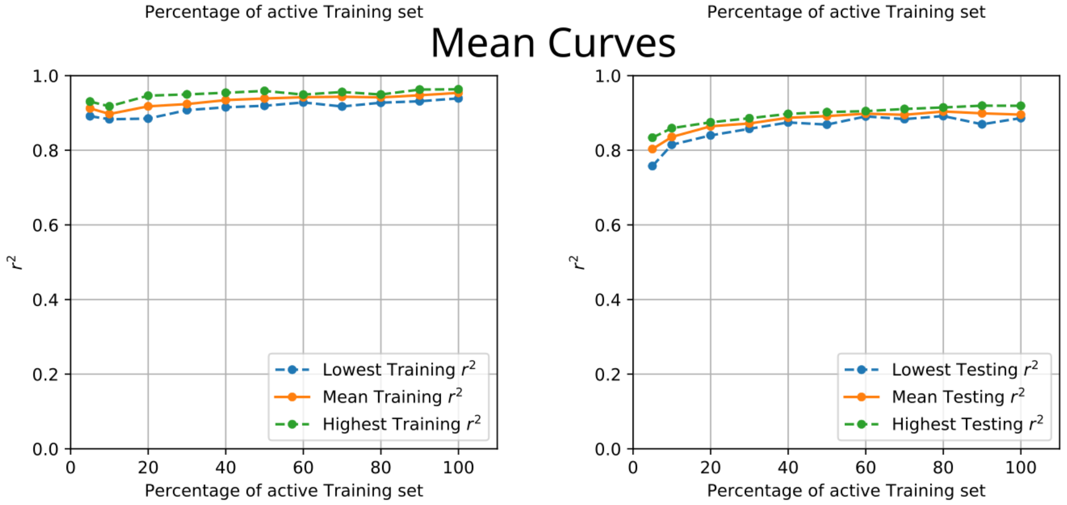
Mean Curves
r²
Percentage of active Training set
Lowest Training $r^2$
Mean Training $r^2$
Highest Training $r^2$
Lowest Testing $r^2$
Mean Testing $r^2$
Highest Testing $r^2$

S4: Distribution of training data

Below the distributions of training data for all properties of interest are shown. Each time, pairs of the oscillator strength and respective excitation energy are plotted. For the triplet excitation energy, the x-axis shows the respective $\Delta E(Q_1)$ excitation energies, instead. Note that the red contour lines in all plots show the kernel density estimation for the scattered data, containing 10, 20, 50 and 95% of the data.

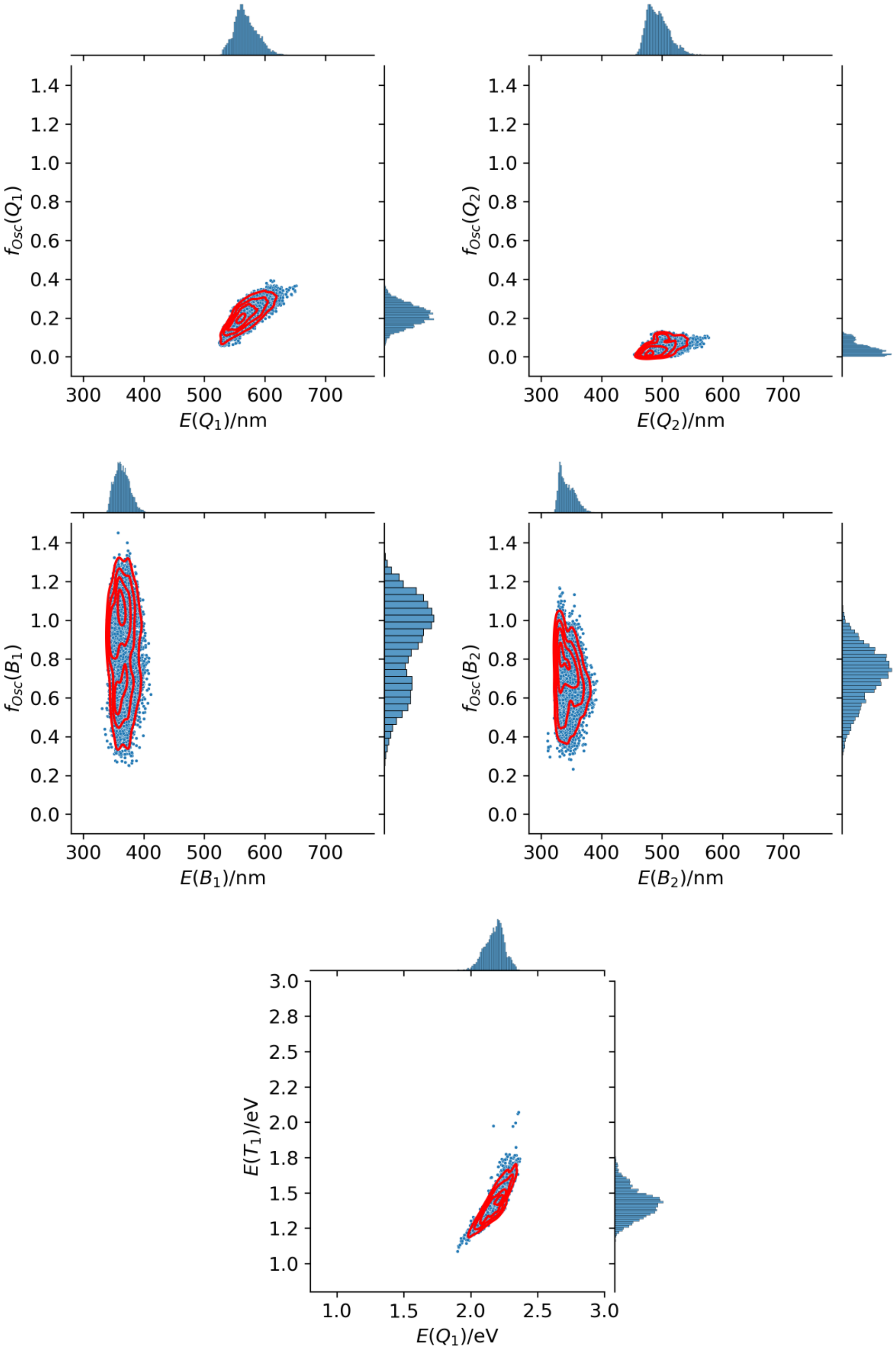

S5: Distribution of prediction data

Below the distributions of prediction data for all properties of interest are shown. Each time, pairs of the oscillator strength and respective excitation energy are plotted. For the triplet excitation energy, the x-axis shows the respective $\Delta E(Q_1)$ excitation energies, instead.

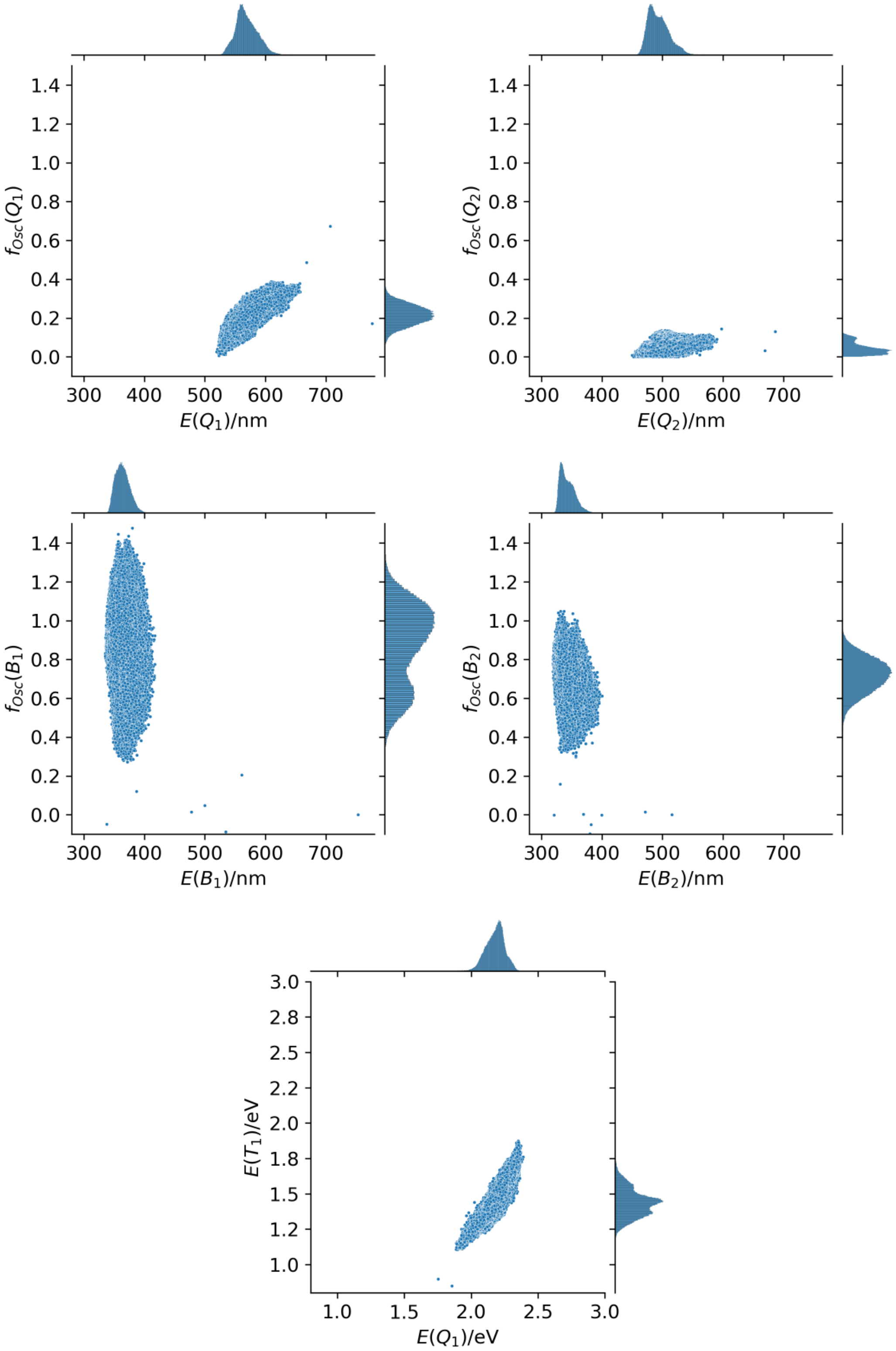

S6: ML procedure, descriptors and ML model applied in this study

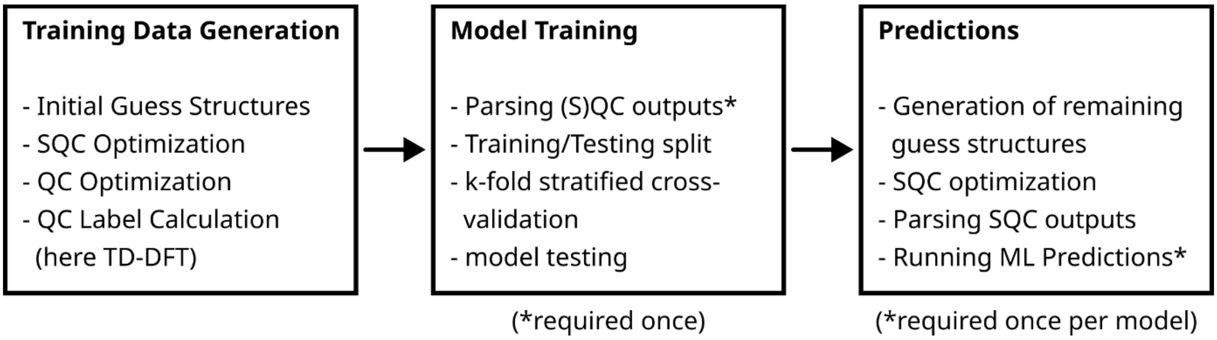


*Figure S1: Overview on the full ML procedure, with the individual steps/calculations involved.*

<u>Model and descriptors</u>

The predictions provided by ArchOnML are based on Kernel Ridge Regression (KRR) model. A KRR model generates a predicted values $E^{pred}$ for an unknown probe molecule M' by utilizing the expression

$$E^{pred}(M',M) = \sum_i \alpha_i f(M',M_i)$$

Here, $\alpha_i$ are the individual weights when determining the similarity (or kernel) function $f$ between the probe molecule and all molecules that the model was trained on contained in the reference space $M$. Weights $\alpha_i$ are obtained through minimizing the expression

$$min\alpha \sum_i \left(E^{pred}(M',M) - E^{label}\right)^2 + \lambda \sum_i \alpha_i^2$$

where $E^{label}$ are the (TD)-DFT values to be trained against, and $\lambda$ is the regularization hyperparameter.

The kernel function used in our specific model is of a gaussian type,

$$f(M',M_i) = e^{-\Sigma_g\left(d_g(M_i,M_j)^2/(2\sigma_g^2)\right)}$$

where $d_g$ is an abstract distance between two molecules $M_i$ and $M_j$ with respect to the $g$-th descriptor. Further, $\sigma_g$ constitues a set of gaussian width hyperparameters that are assigned to each individual descriptor. All hyperparameters are optimized through a two-dimensional grid-based 5-fold cross validation procedure. The two dimensions scanned during cross validation concern the parameter $\lambda$ directly, and a scaling factor $\sigma$ that is applied to initial guess values for $\sigma_g$ simultaneously. For more details on the procedure, please refer to the documentation of the program code. Finally, note that for each desired property, an individual model was trained using the desired property itself as the argument of stratification.

The following list of keywords defines which descriptors are used to train the KRR models. Note that in ArchOnML, requesting a certain descriptor keyword may produce several separate descriptor values belonging to this type, rather than one value per keyword. This is because some types make use of a user-given variable called **MOWin** - a global variable that determines how many semiempirical orbitals will be considered for these descriptor types. For more details, please refer to the documentation of ArchOnML. With the current settings, a total of 50 descriptors are generated.

Finally, since the used ML model is of KRR type, predictions are formulated by comparing unknown probe molecules to a set of reference molecules. Here, the similarity between molecules is then determined in terms of so-called abstract distances, which are either obtained from just forming the

absolute difference of two scalar descriptor values, or by calculation of the Euclidean norm, in case a Coulomb matrix(-like) object is used compare molecules. The original formulation of the latter process can be found in Rupp, M. et al., Phys. Rev. Lett. 108, 058301 (2012).(Rupp et al. 2012) More details on how the similarity between molecules is determined in ArchOnML can be found in the documentation (https://github.com/archonml/archonml).

**SEmpNEl** This descriptor type uses the individual molecule's number of electrons $N_{\mathrm{El}}$, as is.

**SEmpOccs** This descriptor type reads the semi-empirical orbital energies for the occupied orbitals $E$(HOMO−$N$), with $N$ going up to the specified **MOWin**.

**SEmpVirs** This descriptor type is the unoccupied (or virtual) space counterpart of **SEmpOccs**. It thus considers the energies of $E$(LUMO + $M$), with $M$ going up to **MOWin**.

**SEmpEigCoul** This descriptor type generates the eigenvalues of a Coulomb matrix, as originally described in Rupp, M. et al., Phys. Rev. Lett. 108, 058301 (2012), as

$$CM_{IJ} = \begin{cases} 0.5Z_I^{2.4} for I = J \\ \frac{Z_I Z_J}{|R_I - R_J|} for I \neq J \end{cases}$$

Here, matrix elements refer to the $I$-th and $J$-th atoms, where $Z_I$ and $R_I$ refer to the atomic number and position in cartesian space of atom $I$, respectively. The abstract distance between two molecules for this descriptor (and all other Coulomb matrix-like descriptors) is calculated as the aforementioned Euclidean norm between the eigenvalues of two different molecules $M_i$ and $M_j$ according to

$$d_{EigCoul}(M_i, M_j) = \sqrt{\sum_I \left|\epsilon_I^i - \epsilon_I^j\right|^2},$$

where $\epsilon_I$ is the $I$-th eigenvalue of the specific molecule $M_i$ or $M_j$, sorted in descending order. Note, that in case there is a difference in the number of atoms between $M_i$ and $M_j$, the shorter array is supplemented by zeros.

**SEmpOccEigCoul** This descriptor type makes use of a Coulomb matrix-like object, in which the atomic charges of the original Coulomb matrix in the mixing terms are additionally multiplied with the amount of Mulliken charge $q_I$ at each atom $I$.

**SEmpOccPCMEigCoul** This descriptor constructs a Coulomb matrix-like object that additionally considers the so-called $p$-orbital character of an occupied orbital HOMO-$N$ at each specific atom $I$ (or $J$, likewise). Here, $p$-orbital character $p_I(N)$ is quantified abstractly as the sum of coefficients of atomic orbitals' $p$-character at each specific atom for the specific molecular orbital HOMO-$N$, thus becoming an orbital shape descriptor.

$$p_I(N) = p_{I,x}(N) + p_{I,y}(N) + p_{I,z}(N)$$

Here, $p_{I,x}$ gives the sum of all atomic orbital coefficients at atom $I$ of $p_x$ character, and $N$ stands for the HOMO-$N$ orbital. The other cartesian components are summed up and added. Ultimately, the Coulomb matrix-like object is then defined as

$$CM_{IJ}^{p-char}(N) = \begin{cases} 0.5|Z_I p_I(N)|^{2.4} for I = J \\ \frac{(|Z_I p_I(N)|)(|Z_J p_J(N)|)}{|R_I - R_J|} for I \neq J \end{cases}$$

Note that this descriptor is affected by **MOWin**, thus considering occupied orbitals HOMO-$N$ up to $N$ = **MOWin**. When constructing the abstract distances, only identical orbital indices are compared, applying the same sorted Euclidean norm formalism introduced earlier.

**SEmpVirPCMEigCoul** This descriptor is the analogue of **SEmpOccPCMEigCoul**, that considers unoccupied orbitals instead. It also considers multiple orbitals using **MOWin**.

**SEmpHOLUPDiff** This descriptor keyword requests construction of Coulomb matrix-like objects that consider local electron density differences of $p$-orbital type between only the HOMO and LUMO. Here, "$p$-orbital character density difference at atom $I$ with respect to HOMO and LUMO" $D_I$ is calculated as the difference of squared, local $p$-orbital characters according to

$$D_I(M=0,N=0) = p_I(M=0)^2 - p_I(N=0)^2$$

The Coulomb matrix-like object is then defined as

$$CM_{IJ}^{p-diff} = \begin{cases} 0.5|Z_I D_I(M=0,N=0)|^{2.4} for I = J \\ \dfrac{(|Z_I D_I(M=0,N=0)|)(|Z_J D_J(M=0,N=0)|)}{|R_I - R_J|} for I \neq J \end{cases}$$

**SEmpOrbEnDiffs** This descriptor compares semiempirical orbital energy differences in eV between occupied orbital HOMO-$N$ and virtual orbital LUMO+$M$.

$$E_{Diff}(M,N) = E(LUMO + M) - E(HOMO - N)$$

When calculating the abstract distance for a pair of molecules $M_i$ and $M_j$, it will only compare same-index-pairs. In other words, it will only compare the energy differences of a certain HOMO-N to LUMO+M transition in both molecules – but never use different orbital indices.

**SEmpTransPCMEigCoul** This descriptor type abstractly expresses a transition moment between orbitals HOMO-$N$ and LUMO+$M$ by multiplying local $p$-characters of the two orbitals at atoms $I$ and forming a Coulomb matrix-like object out of it. This so-called local transition contribution $T_I(M, N)$ at atom I for orbitals HOMO-$N$ and LUMO+$M$ is then defined as

$$T_I(M,N) = p_I(M) * p_I(N)$$

and the corresponding matrix expression is constructed as

$$CM_{IJ}^{T}(M,N) = \begin{cases} 0.5(|Z_I T_I(M,N)|)^{2.4} for I = J \\ \dfrac{(|Z_I T_I(M,N)|)(|Z_J T_J(M,N)|)}{|R_I - R_J|} for I \neq J \end{cases}$$

Again only same-index pairs for $N$ and $M$ are considered in the Euclidean norm.

**SEmpOccVirPTransSum** This descriptor keyword requests the summation of all atomic contributions of the above-defined $p$-character transition densities at each atom $I$ between orbital pairs HOMO−$N$ and LUMO+$M$ in scalar form according to

$$TS(M,N) = \sum_I |T_I(M,N)|^2.$$

The abstract distance between molecules for this sum of local transitions is obtained as the simple absolute difference of the scalar values.

S7: Criterion for B band state selection in the ML scheme

For the evaluation of the Soret band, the two excited states with the highest oscillator strengths were selected, after exclusion of the $S_1$ (= $Q_y$) and $S_2$ (= $Q_x$) state transitions. To avoid selecting an unrealistic state that has a high $f$, but also a transition energy outside of the B band, an additional selection criterion was based on the cumulative oscillator strength: the sum of all considered states' $f$ was tracked, and once this sum exceeded a value of 4, no further states were considered. This ensured a focused analysis on the most intense transitions contributing to the Soret band, while maintaining a consistent basis for comparison.

S8: Comparison of ML data to conventional QC: TD-DFT (gas phase and acetone)

*Q band shifts*

*Table S1: Vertical excitation energies (in eV) and oscillator strengths of the first electronic transition ($S_0 \rightarrow S_1$) for various Q red-shifted Chls, from different theoretical models. For the artificial Chls, difference to Chl f is given in parentheses.*

| | Model | Chl *f* | Chl *df* | Chl *f+* |
|---|---|---|---|---|
| $\Delta E_{0-S1}$ / eV | Pred. (vac.) | 2.068 | 2.047 (-0.021) | 2.045 (-0.023) |
| | TD-DFT (vac.) | 2.250 | 2.239 (-0.011) | 2.241 (-0.009) |
| | TD-DFT (acetone) | 2.136 | 2.158 (+0.022) | 2.196 (+0.060) |
| $f_{0-S1}$ | Pred. (vac.) | 0.31 | 0.32 (+0.01) | 0.36 (+0.05) |
| | TD-DFT (vac.) | 0.42 | 0.41 (-0.01) | 0.47 (+0.05) |
| | TD-DFT (acetone) | 0.63 | 0.63 (±0.00) | 0.63 (±0.00) |

The gas phase TD-DFT calculations shown in Table S1 agree well with the KRR predictions. However, calculations in acetone imply a sensitivity regarding the first excited state, especially for $\Delta E_{0-S1}$. Instead of shifting slightly down in energy, TD-DFT computes the new variants as shifted up compared to Chl f. Comparing the vacuum and acetone values shows that TD-DFT predicts Chl *f* to be more affected by the presence of the solvent than the suggested Chl variants (about 120 meV shift compared to 80 or 50 meV for the new variants). The actual shift can thus be considered to be dependent on the present environment.

*B band shifts*

*Table S2: Vertical excitation energies (in eV) and oscillator strengths of the selected B band electronic transition ($S_0 \rightarrow S_X$) for various B red-shifted Chls, from different theoretical models. For the artificial Chls, difference to Chl b is given in parentheses. Values in italics correspond to X = 3, as here, the selected X was different; only applies to TD-DFT in gas phase.*

| | Model | Chl *b* | DVChl *b* | Chl *bb* | Chl *bd* |
|---|---|---|---|---|---|
| $\Delta E_{0-SX}$ / eV | Pred. (vac.) | 3.378 | 3.218 (-0.160) | 3.222 (-0.156) | 3.288 (-0.090) |
| | TD-DFT (vac.) | 3.846<br>*3.695* | 3.592 (-0.254)<br>*/ (-0.103)* | 3.583 (-0.263)<br>*/ (-0.112)* | 3.663 (-0.183)<br>*3.548 (-0.147)* |
| | TD-DFT (acetone) | 3.446 | 3.315 (-0.131) | 3.278 (-0.168) | 3.364 (-0.082) |
| $f_{0-SX}$ | Pred. (vac.) | 1.04 | 1.23 (+0.19) | 1.09 (+0.05) | 1.07 (+0.03) |
| | TD-DFT (vac.) | 1.54<br>*0.86* | 1.40 (-0.14)<br>*/ (+0.54)* | 1.49 (-0.05)<br>*/ (+0.63)* | 1.34 (-0.20)<br>*0.85 (-0.01)* |
| | TD-DFT (acetone) | 1.75 | 1.99 (+0.24) | 1.73 (-0.02) | 1.88 (+0.13) |

As noted above, the state identification issue mentioned above and in the main article affects the interpretation of the B band states in Table S2. TD-DFT generally predicts two or more states with significant (more than 0.5) oscillator strengths, and both the predictions and acetone models agree that usually the lowest B band state ($S_3$) is a bright/the brightest state, respectively. The values in italics indicate those cases for which $S_3$ was *not* the one with highest $f$, which only applies to TD-DFT in vacuum. When neglecting the vacuum TD-DFT calculations and their more difficult state assignment, we can see that the ML predictions nicely agree with the TD-DFT/acetone calculations, both for energies and oscillator strengths.

S8, continued

*Triplet-shifted Chl variants*

*Table S3: Vertical energy differences between the $S_0$, $S_1$ and $T_1$ states (in eV) for various Chls with $\Delta E_{0-S1}$ close to that of Chl a, but with shifted triplet energies, from different theoretical models. For the artificial Chls, difference to Chl a is given in parentheses.*

| | Model | Chl *a* | 3Ac-Chl+ | DVChl *bf* |
|---|---|---|---|---|
| $\Delta E_{0-S1}$ / eV | Pred. (vac.) | 2.144 | 2.151 (-0.007) | 2.153 (+0.009) |
| | TD-DFT (vac.) | 2.358 | 2.350 (-0.008) | 2.305 (-0.053) |
| | TD-DFT (acetone) | 2.316 | 2.320 (+0.004) | 2.258 (-0.058) |
| $\Delta E_{0-T1}$ / eV | Pred. (vac.) | 1.358 | 1.298 (-0.060) | 1.499 (+0.141) |
| | TD-DFT (vac.) | 1.389 | 1.308 (-0.081) | 1.501 (+0.112) |
| | TD-DFT (acetone) | 1.395 | 1.334 (-0.061) | 1.493 (+0.098) |

The TD-DFT results of Table S3 agree well with the predictions for the 3Ac-Chl+ case (for both vacuum and acetone), showing minute changes for $\Delta E_{0-S1}$ compared to Chl *a*, and a small shift to lower $\Delta E_{0-T1}$. For DVChl bf, TD-DFT $\Delta E_{0-S1}$ values are slightly lower than predicted, resulting in a downshift where the KRR would predict a rise in energy. $\Delta E_{0-S1}$ still remains close (less 0.06 eV different) to the Chl *a* excitation, which we consider close enough to possibly maintain original Chl *a* photophysics. Consequently, $\Delta E_{0-T1}$ of DVChl *bf* is also computed by TD-DFT to be lower in energy than the ML predictions. We assign this issue of vinyl-containing Chls to be generally red shifted to the vinyl group conformations, as discussed in the main article. Qualitatively, however, TD-DFT calculations agree with the KRR, also quantitatively in the case of 3Ac-Chl+. The solvent is found to be not relevant for the quality of the B band results.

S9: Comparison of ML data to conventional QC: DFT/MRCI

DFT/MRCI calculations for systems of the investigated size are challenging, since even high-end computers struggle with memory problems in several cases. Unfortunately, we were thus unable to obtain any $\Delta E_{0-T1}$ values from DFT/MRCI with the available computing resources. For the other cases, fortunately, we found that gas phase vs. acetone results only differ by less than 0.01 eV. We also restrict the discussion to the energies, as we were unable to obtain the $f$ values consistently due to the computer memory issues mentioned above. Qualitatively we find that DFT/MRCI values agree with the predicted values, though the shifts resulting from DFT/MRCI are quantitatively closer to the TD-DFT results than to the predicted ones. The range of the differences between methods is however below 0.05 eV (Q band) or 0.1 eV (B band). This means that our predictions hold, showing a possible slight red shift for the predicted Q-shifted variants, although this could be dependent on the environment, as indicated by TD-DFT. For the B band, we are confident that the suggested Chl variants should be B band red shifted by about 0.15 eV compared to Chl *b*.

*Table S4: Vertical DFT/MRCI excitation energy differences (in eV) to Chl f or Chl b for several predicted Chl variants, compared to ML-predicted and TD-DFT values (averaged from Table S1 and Table S2 gas phase/acetone calculations).*

| Method | $\Delta\Delta E_{0-S1}$(Target-Chl *f*) / eV | | $\Delta\Delta E_{0-S3}$(Target-Chl *b*) / eV | | |
|---|---|---|---|---|---|
| | Chl *df* | Chl *f+* | DVChl *b* | Chl *bb* | Chl *bd* |
| Pred. (vac.) | -0.02 | -0.02 | -0.16 | -0.16 | -0.09 |
| TD-DFT (avg.) | +0.01 | +0.05 | -0.12 | -0.14 | -0.165 |
| DFT/MRCI | -0.03 | -0.02 | -0.06 | -0.12 | -0.17 |

S10: Example QC inputs during the ML scheme

Input for PM6 pre-optimization (command line only)

```
#T OPT PM6 symmetry=none geom(nodistance,noangle,nodihedral) symmetry=none
```

```
#IOp(2/9=1111, 2/11=2, 4/33=0) Guess(Always)
```

Input for DFT optimization (singlet and triplet) (command line only)

```
#T CAM-B3LYP/6-31G* OPT symmetry=none geom(nodistance,noangle,nodihedral)
```

```
#iop(6/7=2, 4/33=0, 2/9=1111, 2/11=2) 5D 7F
```

Input for TD-DFT calculations (command line only)

```
#p CAM-B3LYP/6-31G* td(nstates=12, root=1) Symmetry=None GFINPUT GFPRINT iop(6/7=3,9/40=5) 5D 7F
```

S11: Predicted values in Figures 3 and 5 of the main article

*Table S5: Predicted excitation energies (E) and oscillator strengths (f) values of the explicitly considered Chl variants in the main article, for the excited states given in the first row. Energies include the shift for better comparison to the experimental maxima/values (Q states: -0.278 eV, B states: -0.615 eV, triplets: -0.019 eV).*

| | $Q_y$ | | $Q_x$ | | $B_x$ | | $B_y$ | | $T_1$ |
|---|---|---|---|---|---|---|---|---|---|
| | E / eV | *f* | E / eV | *f* | E / eV | *f* | E / eV | *f* | E / eV |
| Chl *a* | 2.144 | 0.255 | 2.522 | 0.037 | 3.466 | 0.881 | 3.724 | 0.698 | 1.357 |
| Chl *b* | 2.250 | 0.171 | 2.581 | 0.022 | 3.378 | 1.044 | 3.550 | 0.740 | 1.561 |
| 8OH-Chl | 2.149 | 0.238 | 2.517 | 0.029 | 3.461 | 0.882 | 3.695 | 0.753 | 1.377 |
| Chl *d* | 2.087 | 0.239 | 2.447 | 0.033 | 3.410 | 0.945 | 3.689 | 0.718 | 1.287 |
| Chl *f* | 2.068 | 0.315 | 2.475 | 0.049 | 3.440 | 0.642 | 3.730 | 0.696 | 1.309 |
| 3Ac-Chl | 2.144 | 0.230 | 2.498 | 0.028 | 3.447 | 0.969 | 3.706 | 0.670 | 1.359 |
| DVChl *a* | 2.157 | 0.230 | 2.536 | 0.026 | 3.424 | 1.063 | 3.694 | 0.790 | 1.381 |
| DVChl *b* | 2.237 | 0.148 | 2.588 | -0.004 | 3.218 | 1.234 | 3.419 | 0.619 | 1.536 |
| Chl *df* | 2.047 | 0.324 | 2.326 | 0.080 | 3.390 | 0.553 | 3.689 | 0.519 | 1.220 |
| Chl *f+* | 2.046 | 0.359 | 2.338 | 0.105 | 3.359 | 0.361 | 3.658 | 0.572 | 1.230 |
| Chl *bb* | 2.312 | 0.082 | 2.637 | 0.028 | 3.222 | 1.093 | 3.292 | 0.601 | 1.679 |
| Chl *bd* | 2.186 | 0.175 | 2.488 | 0.018 | 3.288 | 1.073 | 3.510 | 0.716 | 1.457 |
| 3Ac-Chl+ | 2.151 | 0.290 | 2.413 | 0.096 | 3.396 | 0.528 | 3.679 | 0.641 | 1.298 |
| DVChl *bf* | 2.153 | 0.214 | 2.501 | 0.011 | 3.187 | 1.058 | 3.407 | 0.618 | 1.499 |

S12: Initial geometry generation procedure within ArchOnML

To generate the initial guess structures that will be used in the SQC pre-optimization (and DFT optimization, if part of the training set), a stepwise substitution procedure is applied. This procedure first loads the all-hydrogen substituted core structure from the commented xyz file (see section S1), as well as all substitution fragments with their respective, commented xyz files.

Here, in the core structure the comments !X1 and !Y1 mark the carbon and hydrogen atoms that represent substitution site 1 – and the bond between the two atoms dictates the directionality with which the new substituent will be placed, initially. Likewise, the substituents (and all other sites in the core structure) are marked in the same manner, thus also pre-defining the orientation in which to attach the fragments. In the following, these directions will be referred to as attachment vectors, and the atoms marked as X will be called the bridging atoms, whereas the atoms marked as Y will be referred to as dangling atoms.

When presented with a specific fingerprint (e.g. [4, 3, 1] for a molecule with three substitution sites $R_1$, $R_2$, $R_3$), the structure generator loads the respective xyz information of fragment "4" and then calculates the transformation matrix that would result in an anti-parallel alignment of the two attachment vectors using Rodrigues' rotation formula. The resulting transformation matrix is applied to the substitution fragment and the dangling atoms are deleted from both structures.

Next, the bridging atoms need to be placed in a reasonable distance to each other as to form the new bond between the substituent and the core. For this purpose, the generator identifies which bridging atom types are to be connected in this substitution event (e.g. 'C' and 'C') and takes an initial guess distance from a user-defined table.

After actually placing the fragment at the initial bond distance, a quaternion transformation is performed to rotate the fragment around the bond in a number of (user-definable) rotation steps. In this work, 6 rotations at 30° each are evaluated, whenever a fragment is placed. For every such rotation, an artificially exaggerated nuclear repulsion across the whole joint structure is calculated. The potential of this repulsion is defined as

$$V(R_i, R_j) = \begin{cases} \sum_{i \neq j} \frac{Z_i Z_j}{R_{ij}} & \text{for } R_{ij} > 0.6 \\ \\ \sum_{i \neq j} 10 \frac{Z_i Z_j}{R_{ij}} & \text{else} \end{cases}$$

Here, $R_{ij}$ is the distance between atoms $R_i$ and $R_j$ in angstroms, Z gives the atomic charge of the nuclei and the threshold of 0.6 can be defined by the user. This exaggerated potential was found to greatly alleviate issues with light hydrogen atoms landing too close to other atoms during rotation. Ultimately, after generation and evaluation of the differently rotated versions, the joint structure with the smallest overall nuclear repulsion is adopted. From this updated, singly substituted structure, the process is repeated with substitution site 2, and so on, until all substituents are placed.

Note that we are fully aware of the crudeness of the initial guess structures, and that initial structures depend on the specific succession of sites – but application of more sophisticated methods (like force-fields of molecular dynamics) for the initial guess generation would already diminish the achievable speed-up of performing an ML-assisted screening in the first place, since all initial guess structures will always be subject to at least the SQC pre-optimization treatment, anyways.

S13: 3D structures for candidates of interest

Below, the 3D structures of the six candidates are provided. Note, that all shown structures are depicted as seen from diagonally above and rotated by 90° clockwise with respect to their Lewis structures as seen in Figure 5. This viewing angle was chosen such that the placeholder sidechains X and Y (see Figure 1) do not block the view on the substituent sites.

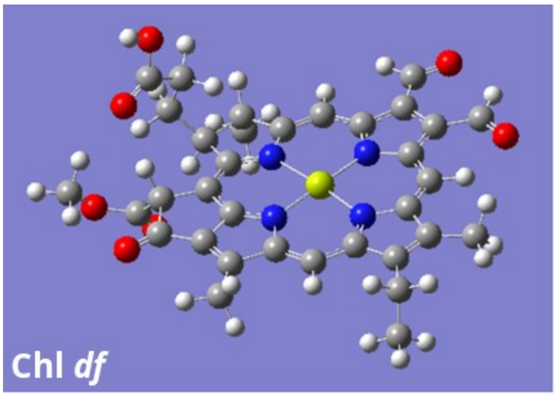


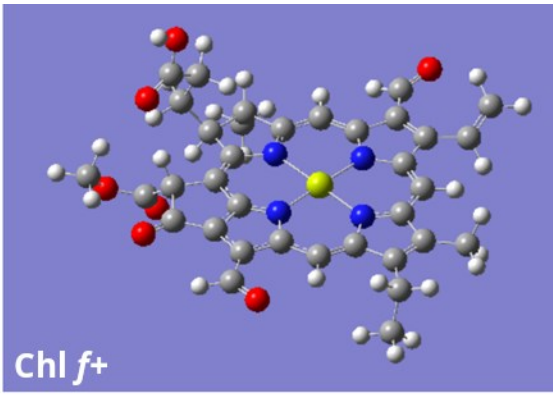


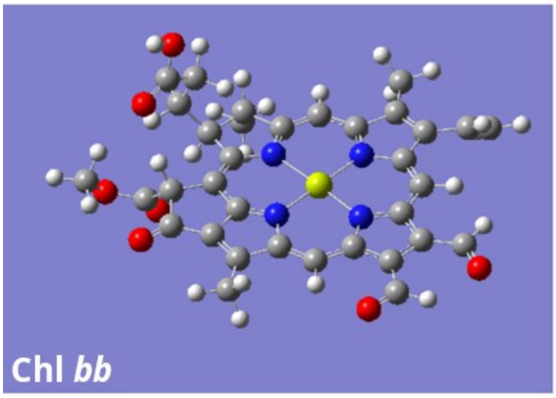


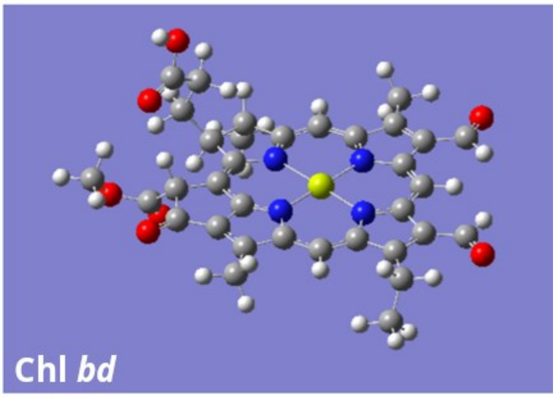


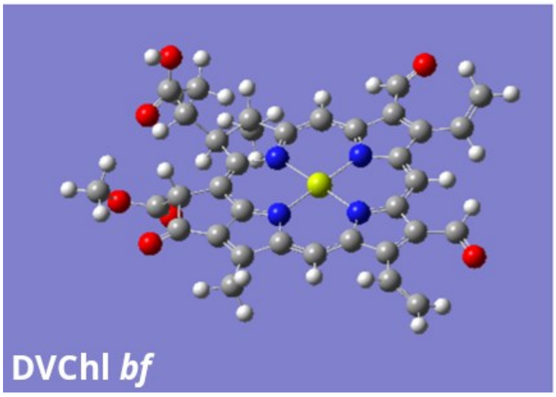


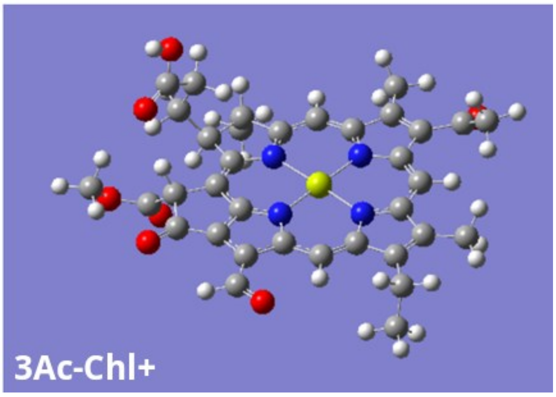


**References**

Rupp M, Tkatchenko A, Müller K-R, von Lilienfeld OA (2012) Fast and Accurate Modeling of Molecular Atomization Energies with Machine Learning. Phys Rev Lett 108:058301. https://doi.org/10.1103/PhysRevLett.108.058301
